# MSH3 Polymorphisms and Protein Levels Affect CAG Repeat Instability in Huntington's Disease Mice

**DOI:** 10.1371/journal.pgen.1003280

**Published:** 2013-02-28

**Authors:** Stéphanie Tomé, Kevin Manley, Jodie P. Simard, Greg W. Clark, Meghan M. Slean, Meera Swami, Peggy F. Shelbourne, Elisabeth R. M. Tillier, Darren G. Monckton, Anne Messer, Christopher E. Pearson

**Affiliations:** 1 Genetics and Genome Biology, The Hospital for Sick Children, Toronto, Ontario, Canada; 2 Wadsworth Center, New York State Department of Health, Albany, New York, United States of America; 3 Department of Biomedical Sciences, University at Albany, SUNY, Albany, New York, United States of America; 4 Department of Medical Biophysics, University of Toronto, Toronto, Ontario, Canada; 5 Campbell Family Institute for Cancer Research, Ontario Cancer Institute, University Health Network, Toronto, Ontario, Canada; 6 Department of Molecular Genetics, University of Toronto, Toronto, Ontario, Canada; 7 Institute of Molecular, Cell, and Systems Biology, College of Medical, Veterinary, and Life Sciences, University of Glasgow, Glasgow, United Kingdom; Stanford University School of Medicine, United States of America

## Abstract

Expansions of trinucleotide CAG/CTG repeats in somatic tissues are thought to contribute to ongoing disease progression through an affected individual's life with Huntington's disease or myotonic dystrophy. Broad ranges of repeat instability arise between individuals with expanded repeats, suggesting the existence of modifiers of repeat instability. Mice with expanded CAG/CTG repeats show variable levels of instability depending upon mouse strain. However, to date the genetic modifiers underlying these differences have not been identified. We show that in liver and striatum the R6/1 Huntington's disease (HD) (CAG)∼100 transgene, when present in a congenic C57BL/6J (B6) background, incurred expansion-biased repeat mutations, whereas the repeat was stable in a congenic BALB/cByJ (CBy) background. Reciprocal congenic mice revealed the *Msh3* gene as the determinant for the differences in repeat instability. Expansion bias was observed in congenic mice homozygous for the B6 *Msh3* gene on a CBy background, while the CAG tract was stabilized in congenics homozygous for the CBy *Msh3* gene on a B6 background. The CAG stabilization was as dramatic as genetic deficiency of *Msh2*. The B6 and CBy *Msh3* genes had identical promoters but differed in coding regions and showed strikingly different protein levels. B6 MSH3 variant protein is highly expressed and associated with CAG expansions, while the CBy MSH3 variant protein is expressed at barely detectable levels, associating with CAG stability. The DHFR protein, which is divergently transcribed from a promoter shared by the *Msh3* gene, did not show varied levels between mouse strains. Thus, naturally occurring MSH3 protein polymorphisms are modifiers of CAG repeat instability, likely through variable MSH3 protein stability. Since evidence supports that somatic CAG instability is a modifier and predictor of disease, our data are consistent with the hypothesis that variable levels of CAG instability associated with polymorphisms of DNA repair genes may have prognostic implications for various repeat-associated diseases.

## Author Summary

The genetic instability of repetitive DNA sequences in particular genes can lead to numerous neurodegenerative, neurological, and neuromuscular diseases. These diseases show progressively increasing severity of symptoms through the life of the affected individual, a phenomenon that is linked with increasing instability of the repeated sequences as the person ages. There is variability in the levels of this instability between individuals—the source of this variability is unknown. We have shown in a mouse model of repeat instability that small differences in a certain DNA repair gene, MSH3, whose protein is known to fix broken DNA, can lead to variable levels of repeat instability. These DNA repair variants lead to different repair protein levels, where lower levels lead to reduced repeat instability. Our findings reveal that such naturally occurring variations in DNA repair genes in affected humans may serve as a predictor of disease progression. Moreover, our findings support the concept that pharmacological reduction of MSH3 protein should reduce repeat instability and disease progression.

## Introduction

At least 14 neurodegenerative and neuromuscular diseases are caused by expansions of CAG/CTG repeats including Huntington's disease (HD) and myotonic dystrophy type 1. An inverse correlation between the length of CAG repeat tracts and age-of-onset is observed in HD families [1], [2]. The expanded CAG repeat is unstable in several organs, undergoing progressive length increases over time, coincident with disease progression [3]–[7]. Within the brain, somatic CAG expansions are region-specific with the greatest instability observed in striatum and cortex, which show the most severe neuropathology in HD patients [3]–[7]. The potential contribution of somatic repeat instability to HD/DM1 disease age-of-onset, severity and progression [1], [7], [8], make it imperative to understand the process of instability as it is a therapeutic target [9].

Several transgenic mouse models have contributed to our understanding of the mechanisms of CAG/CTG instability [10]–[18]. Both *cis*-elements and *trans*-factors that modify CAG/CTG repeat instability have been identified. *Cis*-elements include flanking sequence context such as CTCF binding sites, CpG-methylation, DNA sequence, G+C-content, DNA replication direction and progression, and transcription levels and direction [9], [11], [17]–[23]. *Trans*-factors that have been linked to CAG/CTG instability include DNA replication, repair and recombination proteins. Of those tested in mice, *Fen1*, *Rad52*, *Rad54*, *Xpc*, appear to have no effect [24]–[27], while *Ogg1*, *Neil1*, *Csb*, *Lig1*, *Xpa*, *Msh6*, or *Pms2* show partial effects [16], [28]–[33]. However, the mismatch repair (MMR) genes *Msh2* and *Msh3* have been shown to be absolutely required for repeat expansions [13], [16], [26], [27], [34]–[36].

In addition to being the strongest modifiers of repeat instability identified to date, [9], [37] the roles of the MMR proteins MSH2 and MSH3 have recently been extended to CAG/CTG instability in human HD and DM1 stem cells [38]. MMR is a pathway dedicated to protecting against mutations arising from mispaired nucleotides and insertion/deletion loops [39]. There are two heterodimeric protein complexes that recognize unpaired DNAs: MutSα consists of MSH2-MSH6, and MutSβ is formed by MSH2–MSH3. MutSα is predominantly required to repair base-base mismatches, and MutSβ, with some functional redundancy with MutSα, is predominantly involved in the repair of insertion/deletion loops (1-12 nucleotides) [40]–[44]. MutSβ, more so than MutSα, is required to repair short CAG/CTG slip-outs [42]. Recent evidence revealed that the levels of MSH2, MSH3, and MSH6 protein varied widely between 14 different murine tissue types, and MSH3 protein levels were greater than MSH6 levels in most tissues analyzed [45]. MMR typically functions to protect against mutations; however, in the case of long CAG/CTG repeat alleles, MSH2 and MSH3 are required for additional repeat expansion mutations [37]. *Msh2* deficiency stabilized CAG/CTG repeat tracts from inherent expansions in somatic tissues of R6/1 mice transgenic for exon 1 of the HD gene [13], [46], *Hdh^Q111^* knock-in mice [27], [36], and several DM1 mouse models [16], [26], [47]. The MSH3 protein, like MSH2, is required for the expansion-biased CAG/CTG repeat instability in somatic tissues [16], [27], [35]. The absence of *Msh3* blocks CAG/CTG expansions in tissues from HD mice [16], [27], [35]. The absence of one *Msh3* allele (*Msh3*/null mice) is sufficient to decrease CAG expansion frequencies in HD and DM1 mice, suggesting that MSH3 may be a limiting factor in the process leading to the formation of expansions, and that CAG instability could tightly depend on MSH3 protein levels [16], [27], [35]. An absence of MSH6 increased CAG/CTG expansions [16], [27], [35], probably due to the competition between MSH3 and MSH6 for binding to MSH2 to form functional complexes [16], [48].

Several models have been proposed through which MutSβ can drive CAG/CTG expansions. *In vivo* mouse models suggest that MutSβ is required to drive CAG expansions [13], [27], [35] and to protect against repeat contractions [16], [26], [27], [34]–[36], [49]. The role of MutSβ in expansions extends beyond its ability to bind slipped-DNAs [50] as an ATPase-functional MutSβ complex is necessary for CAG expansions [47] and downstream mismatch repair proteins, like PMS2 are partially required for instability [28]. The MutSβ complex may act on CAG repeats during errors at replication forks or during transcription, as both processes can enhance instability in a MMR-dependant manner [51], [52]. Instability in non-proliferating tissues may arise when attempted repair events by MutSβ on clustered short CAG/CTG slip-outs is arrested [42]. Arrested repair along these clusters may allow for strand displacement, slippage, further out-of-register mispairing, and repair synthesis resulting in expansions on un-repaired clustered slip-outs [42]. Reiterations of such events, using the aberrant repair products as substrates, could lead to continuous expansions. Perturbed levels of MutSβ decreased repair of short CTG slip-outs, allowing them to be integrated as expansions [42]. The sensitivity of short TNR slip-out repair to MutSβ concentration is similar to other reports of repair protein levels affecting repeat instability [53]–[55].

In this study we used the R6/1 HD transgenic mouse model [10], [56]. The R6/1 HD transgenic mice were generated by using a construct with ∼1000 bp of the human Huntingtin gene *HTT* promoter, the entire *HTT* exon-1, including ∼116 CAG repeats, and 262 bp of *HTT* intron-1 [56]. The R6/1 transgene has been reported to be integrated as a head to tail dimer on chromosome 3 [57]. However, in our colony the transgene appears to harbour only a single CAG repeat tract length as assessed by SP-PCR (see below). The transgene expresses the expanded CAG transcript and is translated to produce a HTT exon 1 fragment with an expanded polyglutamine tract. Males show limited CAG instability upon transmission and females are infertile, as reported [10], [56]. R6/1 mice have been used extensively to assess both HD pathogenesis and CAG instability, where the latter results have found tissue-specific instability dependent upon *Msh2* and *Msh3* [13], partially-dependent upon *Ogg1* and *Neil1* [31]. R6/1 mice have also been found to be protected from instability by *Csb*, and unaffected by *Fen1* [25], [32]. Furthermore, CAG instability in R6/1 mice has been shown to be sensitive to transcription progression [58] and tissue-specific stoichiometric levels of base excision repair proteins [54].

Several studies have reported the existence of other modifiers of CAG/CTG repeat instability, as different mouse strains harbouring the same HD or DM1 CAG/CTG transgene have variable levels of repeat instability [16], [59], [60]. Similarly, extreme repeat changes in some Huntington's families suggests the existence of family-specific instability modifiers that may be heritable [61]. However, none of these studies have proposed a candidate factor as a source for strain-specific variations in CAG/CTG instability patterns. Here we have identified the source of variable CAG repeat instability between two inbred mouse strains, C57BL/6J (B6) and BALB/cByJ (CBy), congenic for an *HTT* exon 1 transgene (R6/1). Using both congenic and reciprocal congenic mice, we identified coding variations in the *Msh3* gene as sources of the variable levels of somatic CAG instability in the different strains of R6/1 transgenic mice. The B6 MSH3 protein variant is highly expressed and associated with expansion biased mutations, while the CBy MSH3 protein variant is expressed at low levels and is associated with CAG tract stability.

## Results

### CAG repeat instability in C57BL/6J and BALB/cByJ mice

**Figure 1:**
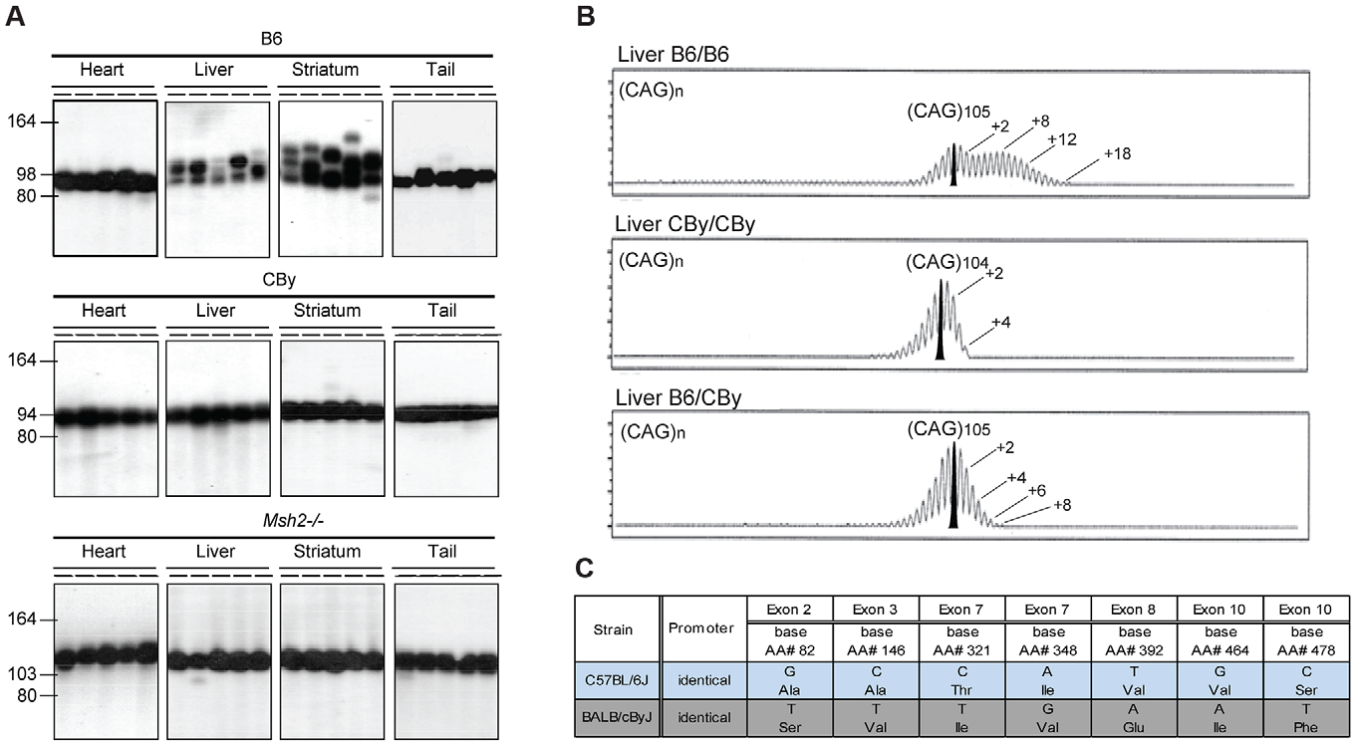
Representative CAG repeat distributions, and *Msh3* variations in B6 and CBy mice. A) The autoradiographs show representative SP-PCR analyses of DNA, extracted from heart, liver, striatum and tail. At weaning the B6.Cg-R6/1 (B6) and CBy.Cg-R6/1 (CBy) congenic mice contained in tail DNA (CAG)98 and (CAG)94, respectively. For comparison the profiles of the *Msh2*−/− mouse is shown. About 5–10 DNA amplifiable molecules were amplified in each reaction with primers MS-1F and MS-1R. Animals were 20-weeks old. B) Congenic CBy.Cg-R6/1 mice were crossed to B6 and the resulting F1 progeny were crossed to produce F2 mice with all possible genotypes at the *Msh3* locus. Repeat instability was assayed by amplifying 10 ng genomic DNA using fluorescently labelled primers and resolving the fragments by capillary gel electrophoresis (Figure 1B). Using this high-resolution approach repeat length distributions present with the typical ‘hedgehog’ pattern (*e.g.* [10], [13], [15], [16]. This pattern reflects both somatic mosaicism within the sample and PCR artefacts generated by *Taq* polymerase slippage [62], [63]. The PCR artefacts are predominantly repeat contractions, hence these are not considered here. The pattern of CAG repeat instability depended on genotype at the MSH3 locus. B6 homozygosity resulted in the greatest instability, CBy homozygosity resulted in lack of expansion, while heterozygosity resulted in an intermediate instability, indicative of a gene dosage effect of the *Msh3* locus. Numbers indicate the CAG repeat size corresponding to major peaks. In addition, on the B6 tracing, a second number indicates the highest CAG repeat number detected. C) *Msh3* polymorphisms in *Msh3* gene from C57BL/6 (B6) and BALB/cBy (CBy) mice. Promoters were identical. SNPs were identified or confirmed to those in *dbSNP* by sequencing the *Msh3* gene.

To assess CAG repeat instability in mice with different genetic backgrounds, we backcrossed B6CBA-Tg(HDexon1)61Gpb(R6/1) transgenic mice [10] to B6 and CBy inbred mice to obtain B6.Cg-Tg(HDexon1)61Gpb (B6.Cg-R6/1) and CBy.Cg-Tg(HDexon1)61Gpb (CBy.Cg-R6/1) congenic lines, respectively. These congenic lines were typed at each generation for the presence of the R6/1 transgene, thus after 10 backcross generations, it was predicted that 99.8% of the genome was homozygous for the inbred line (B6 or CBy), while the remaining 0.2% of the genome remained heterozygous. The B6.Cg-R6/1 and CBy.Cg-R6/1 congenic mice contained (CAG)98 and (CAG)94, respectively - so these mice and their progeny should be well matched for HD transgene effects with the same flanking *cis*-elements. Genome-wide SNP analysis confirmed that the *HTT* transgene had integrated into chromosome 3 [57] and showed minimal contamination of adjacent regions in the congenic strains (Figure S1A, Table S1). We analysed CAG instability by SP-PCR in liver, striatum, tail and heart from 20 week-old mice. B6.Cg-R6/1 mice showed a high level of somatic instability biased toward expansions in liver and striatum (Figure 1A), while the repeat was relatively stable in heart and tail, as previously described [10], [13]. Surprisingly, the CAG repeats were very stable in all of these four tissues from age-matched CBy.Cg-R6/1 mice, including in liver and striatum (Figure 1A). The stabilizing effect of the CBy background was as striking as the genetic deficiency of the MMR protein MSH2, as previously described [13]. Thus, the level of somatic CAG expansions can be dramatically different between B6.Cg-R6/1 and CBy.Cg-R6/1 mice, revealing that CAG expansions are affected by genetic background.

### CAG repeat instability difference is likely a single-gene/locus effect

Towards identifying modifiers of CAG instability, we performed a F2 intercross between CBy.Cg-R6/1 and B6, and tested offspring for differential CAG instability patterns in the liver as this tissue displayed considerably different patterns of CAG instability between the R6/1 congenic lines (Figure 1A). Repeat instability was assayed blind (to remove bias) by high-resolution capillary gel electrophoresis where repeat length distributions present a typical ‘hedgehog’ pattern (*e.g.* [10], [13], [15], [16]) (Figure 1B). In R6/1 mice the overall level of somatic instability is generally relatively low and the inherited or progenitor allele is usually defined as the modal allele within the distribution of peaks (see bold-filled peak in Figure 1B) and is conserved between tissues from the same mouse. As *Taq* polymerase slippage during PCR generates repeat contractions [62], [63], we concentrated on defining an instability phenotype based on the expanded alleles. Using this approach, three distinct patterns of CAG instability in liver DNA of F2 mice were observed (Figure 1B). Firstly, as in the parental B6.Cg.R6/1 mice, some F2 mice presented with high levels of instability with a broad bimodal distribution profile with a second peak at ∼+7–9 repeats and a long tail extending out to greater than +15 repeats. Secondly, as in the parental CBy.Cg.R6/1 mice, some F2 mice presented with only very low levels of CAG mosaicism with a unimodal negatively skewed distribution with a tail of expanded alleles that extended only to +3 or +4 repeats and ended very abruptly. Thirdly, we detected an intermediate instability phenotype in which the distributions were unimodal, but more normally distributed, without the pronounced negative skew, and in which the tail of expansions extended out to +7 to +8 repeats (Figure 1B). These distinct patterns of CAG instability between F2 offspring suggested that they may contain varying dosages of a specific modifier gene(s) of CAG instability. Of 81 mice assessed, 20 had highly unstable, 24 stable and 37 intermediate levels of CAG repeat instability. This phenotypic distribution fits with the 1∶2∶1 segregation ratio expected for a single modifier gene with a semi-dominant allele (Chi-Square analysis (*X^2^*_(2, N = 81)_ = 1.0, *p* = 0.61)).

Since *Msh3* is one of the strongest known drivers of CAG expansions [9], [37], and it also shows a gene dosage effect [16], [27], [35], we considered the possibility that the *Msh3* gene variants between the CBy and B6 mouse strains may account for the variations in CAG instability patterns between the congenic strains. Towards this end, we genotyped the locus containing the *Msh3* gene using microsatellite markers flanking the gene (D13Mit159 and D13Mit147) in the offspring of the F2 intercross. All mice showing high levels of CAG expansions were homozygous for B6 alleles at the *Msh3* locus, while those with the stable CAG tract were homozygous for CBy alleles at the *Msh3* locus and those with the intermediate CAG instability were heterozygous at the *Msh3* locus. These data firmly link variation in *Msh3* or a nearby gene on mouse chromosome 13 with the differential repeat instability phenotypes (LOD score = 48.8, (θ = 0), see Materials and Methods).

### *Msh3* polymorphisms between mouse strains

In an effort to identify *Msh3* gene polymorphisms, we sequenced the exons and promoter of the *Msh3* gene of the CBy and B6 strains. We identified 7 polymorphisms that resulted in non-synonymous amino acid changes, within exons 2, 3, 7, 8 and 10 (Figure 1C), between B6 and CBy. There was no sequence variation of the *Msh3* promoter between the CBy and B6 mice. The polymorphic, coding *Msh3* variants between the CBy and B6 mice may therefore be responsible for the variable CAG instabilities between the mouse lines. It is highly unlikely that the original non-synonymous polymorphisms were unlinked and became linked during the course of the construction of the inbred lines, as we sequenced the *Msh3* gene of the strains from colonies originating independent from those used in our breedings.

### Somatic CAG instability in *Msh3* locus reciprocal congenic mice

**Figure 2:**
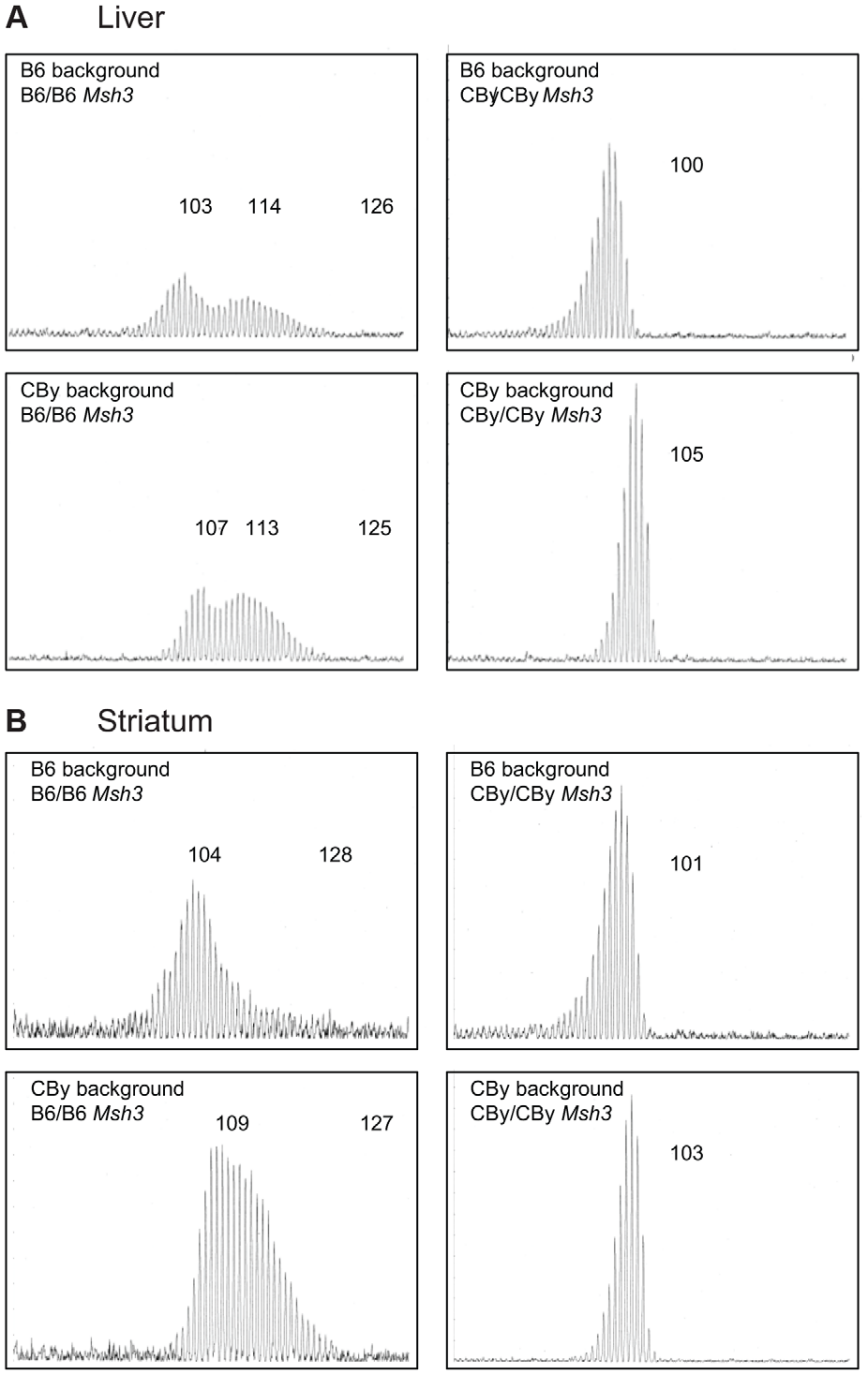
Representative CAG repeat distributions from reciprocal *Msh3* congenic lines of mice. Typical GeneScan traces for sizing of the CAG repeat as outlined in Figure 1B. Liver (A) and Striatum (B) from 16–20 week old R6/1 transgenic mice showing the effect of homozygosity at the *Msh3* locus on the pattern of expansion in the reciprocal congenic mice. Regardless of genetic background, CBy homozygosity at the congenic locus results in loss of somatic expansion, while B6 homozygosity is permissive of somatic expansion.

In order to test the potential role of MSH3 protein variants on CAG instability, we created *Msh3-*locus reciprocal congenic mice carrying the B6 *Msh3* variant on a CBy genetic background (CBy.B6-msh3^B6/B6^), and a CBy *Msh3* variant in the B6 genetic background (B6.CBy-msh3^CBy/CBy^). Each line was backcrossed to the recipient strain 10 times as in the creation of the R6/1 congenic lines. Next they were inter-crossed as appropriate with the R6/1 congenic lines to create mice CBy homozygous at the *Msh3* locus on a B6 genetic background and hemizygous for the R6/1 transgene (B6.CBy-msh3^CBy/CBy^, R6/1) and mice B6 homozygous at the *Msh3* locus on a CBy genetic background and hemizygous for the R6/1 transgene (CBy.B6-msh3^B6/B6^, R6/1). With these mice we could better isolate the effect of each *Msh3* variant on both mouse backgrounds on CAG stability. Genome-wide SNP genotyping revealed minimal donor haplotype contamination in the reciprocal congenic strains B6.CBy-msh3^CBy/CBy^ and CBy.B6-msh3^B6/B6^ and their corresponding R6/1 congenic strains B6.Cg-R6/1 and CBy.Cg-R6/1 (Figure S1B, Table S1). Outside of the genomic region flanking chromosome 3 integration site of the R6/1 transgene [57], there appears to be no contamination of donor DNA in the CBy background line, and only minor areas of residual heterozygosity in the B6 background lines on chromosomes 6, 15 and 17. The contaminating regions linked to the *Msh3* gene in the reciprocal congenics contain a limited number of genes (Figure S7), none of which have an obvious or documented role in CAG repeat instability. The regions linked to the *Msh3* gene in the CBy.B6-Msh3 R6/1 reciprocal congenic mice span 43 Mbp and include 314 genes, of which 233 are protein-coding (Figure S7). In the B6.CBy-Msh3 R6/1 strain, the linked genes cover a region of approximately 22 Mbp, which lies within the 43 Mbp region of the CBy.B6-Msh3 R6/1 strain. A total of 151 genes are found within this region with 104-protein coding transcripts (Figure S7). Therefore, differences in CAG instability between and within the strains were interpreted to be a consequence of the introgressed *Msh3* allele variants. At 16–20 weeks of age, a high level of CAG expansion was present in the liver from mice containing the B6 *Msh3* gene for both B6 and CBy backgrounds. This instability was evident as a broad bimodal distribution profile whereas the liver DNA from mice with the CBy *Msh3* gene showed a low level of instability with a unimodal distribution (Figure 2A). A similar pattern of CAG instability in the striatum further indicated greater levels of CAG instability in mice with the B6 *Msh3* gene than those with the CBy *Msh3* gene (Figure 2B). The striking differences in the levels of instability between mice harbouring B6 *Msh3* compared to CBy *Msh3*, regardless of background, supports the concept that the B6 *Msh3* gene variant drives CAG expansions to a greater degree than does the CBy *Msh3* gene variant.

We also assessed CAG repeat instability in testes and sperm from 12-week old and 24-week old mice (Figure S2). The CAG repeats were relatively stable in the germline of both mouse lines, regardless of age, consistent with the relatively low levels of transmitted mutations observed in our colony and consistent with previous reports of R6/1 mice [10], [13]. A few changes of a single repeat unit were observed in the testes of 24-week old B6 and a similar range was observed in the SP-PCR analysis of sperm DNA. These small changes were not obviously observed in the germline of CBy mice (Figure S2). However, the R6/1 transgenic mice from which the CBy.Cg-R6.1 line was derived initially had ∼115 CAG repeats which decreased to ∼95 repeats over the course of ∼12 years of transmissions (not shown). This observation is consistent with a tendency for CAG contractions to occur in the presence of reduced levels of MMR proteins [16], [26], [27], [34]–[36], [49]. Typically, the R6/1 line gives rise to occasional expansions of 1–2 repeat units/transmission and rarer large contractions [64].

### MSH3, but not MSH2 or MSH6, protein levels are *Msh3* gene variant-dependent

**Figure 3:**
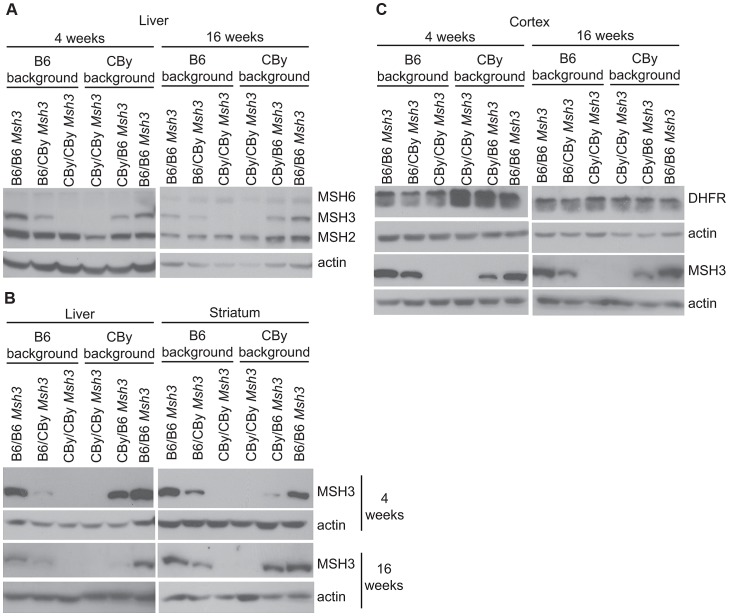
Western blot analysis of MMR and DHFR protein levels. MMR expression in liver and striatum from 4 and 16 week-old mouse. Actin was used as a loading control. MSH2: 104 kD, MSH6: 160 kD, MSH3: 127 kD (Ab = 2F11) and actin: 42 kD. DHFR expression in cortex from 4 and 16 week-old mice DHFR: 21 kD. A) Simultaneous Western blot using MSH2-, MSH3-, MSH6- and actin-specific antibodies in liver. For antibody dilutions see Materials and methods. B) Western blot using only anti-MSH3 (Ab = 2F11) and actin antibodies in liver and striatum. C) Western blot analysis of DHFR in cortex from 4 and 16 week-old mice.

To test the possibility that *Msh3* polymorphisms may affect the expression of MMR proteins, which subsequently lead to variable levels of CAG instability between mouse strains, we assessed MMR protein levels in mouse tissues by Western blotting [38], [42], [45]. In liver, the levels of MSH2 and MSH6 were similar between all mouse strains (Figure 3A). However, the level of MSH3 protein varied widely between mice, with high expression in mice carrying the B6 *Msh3* gene, and undetectable levels in mice carrying the CBy *Msh3* gene (Figure 3A). An intermediate level of MSH3 was reproducibly observed in mice heterozygous for the B6 and CBy *Msh3* genes, on both B6 and CBy genetic backgrounds, thus indicating a gene dosage effect between *Msh3* variant alleles. This pattern did not vary with age (Figure 3A; compare 4 weeks with 16 weeks). The same MSH3 expression patterns were observed using a MSH3-specific antibody alone (Figure 3B, right panel). The striatum displayed the same strain-specific MSH3 expression pattern, where mice homozygous for the B6 *Msh3* gene showed the highest levels of MSH3 protein, while mice homozygous for the CBy *Msh3* gene expressed the lowest level, and mice heterozygous for the *Msh3* allele displayed intermediate MSH3 protein expression (Figure 3B, right panel). It is notable that MSH3 levels varied in a manner that depended on the *Msh3* variant and was independent of mouse strain background. The spleen, thymus, cortex and cerebellum also showed a similar *Msh3* gene variant-specific pattern of MSH3 protein expression (Figure S3). Towards ensuring that the apparent expression variations were not due to differential ability of the antibody to recognize its epitope, we analyzed MSH3 protein expression using an independent monoclonal MSH3 antibody (5A5, which recognizes an epitope within exon 4 compared to 2F11 which recognizes an epitope in exon 1, neither of which have amino acid differences between B6 and CBy mice), as described by [65]. We observed the same expression patterns, suggesting that the MSH3 levels observed in tissues are independent of the binding site of the antibody on MSH3 (Figure S4). Thus, regardless of genetic background, the level of MSH3 protein expression depended upon whether the mouse carried the B6 *Msh3* variant (high) or the CBy *Msh3* variant (low).

### DHFR expression in *Msh3* locus reciprocal congenic mice

The *Msh3* and dihydrofolate reductase (*Dhfr*) genes are arranged in a head-to-head orientation and share a common promoter that divergently drives transcription [66]–[68]. The levels of both transcripts are produced at similar levels in various mouse tissues [67], [68]. We analyzed DHFR expression (Figure 3C) from R6/1 congenic and *Msh3* locus reciprocal congenic mice carrying either homozygous B6 *Msh3* variants, or CBy *Msh3* variants, or B6/CBy variants. DHFR protein levels did not vary between mouse strains, unlike the MSH3 protein (Figure 3C). These results suggest that the variation of MSH3 protein levels between the B6- and CBy-*Msh3* gene variants are not regulated by promoter, which is identical between variants (Figure 1C), but by the coding variations of the *Msh3* gene.

### MSH3 expression in different mouse strains

**Figure 4:**
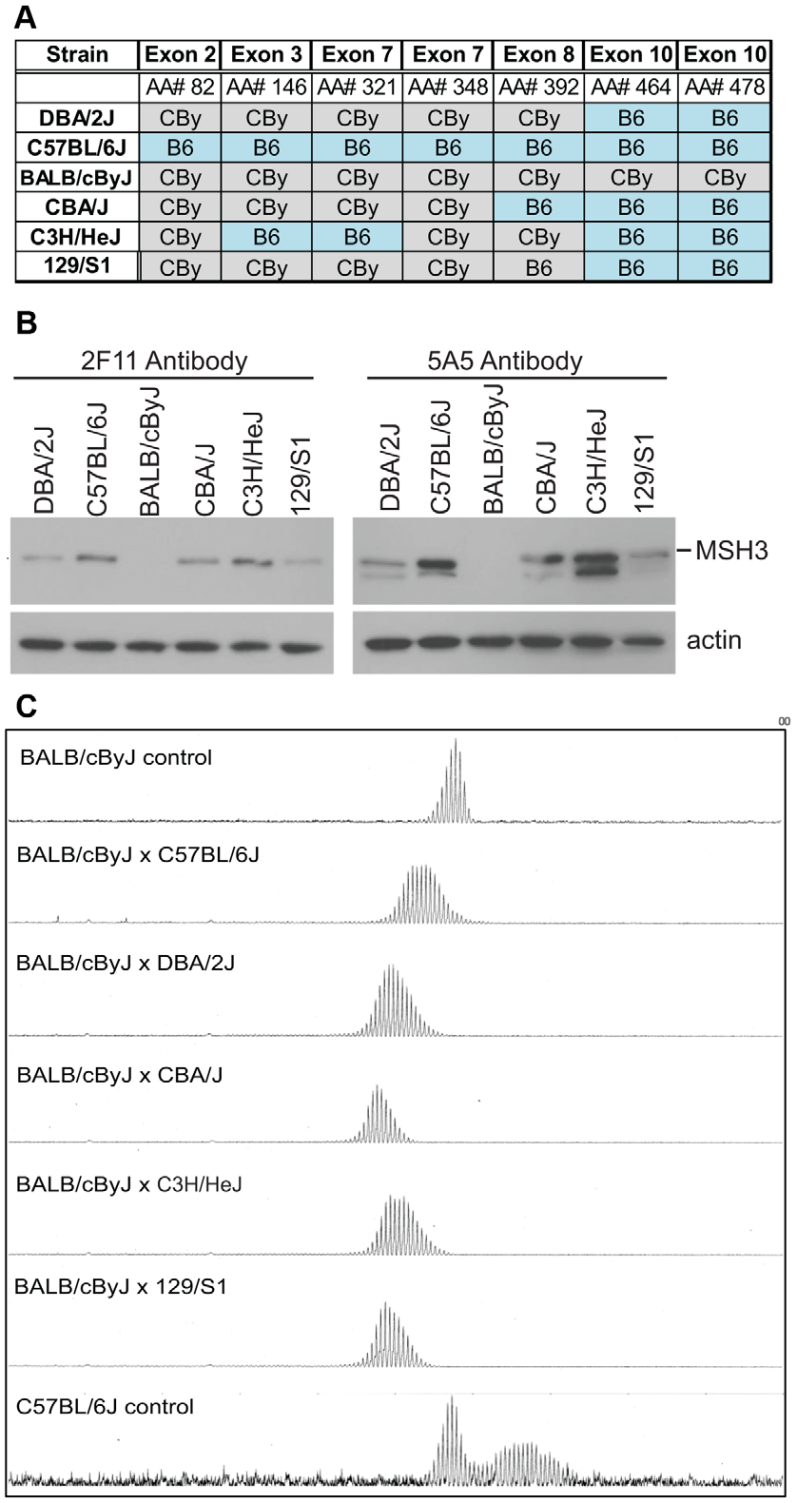
MSH3 coding polymorphisms and protein expression in different mouse strains. A) *Msh3* polymorphisms in *Msh3* gene from C57BL/6 (B6) and BALB/cBy (CBy) mice. Promoters were identical. SNPs were identified or confirmed to those in *dbSNP* by sequencing the *Msh3* gene. In DBA/2J, exon 8, AA#392 was correctly identified to be T/Valine. For a given amino acid the same codon was used for the variants. The complete set of MSH3 protein polymorphisms in 14 mouse strains is in Table S2. B) MSH3 expression in spleen extract from different background using two different MSH3 antibodies [65]. The faster migrating band for 5A5 was a non-specific cross-reacting product, as described for 5A5 but not 2F11 [65]. All other figures in this study used 2F11. C) Typical GeneScan traces for sizing of the CAG repeat as outlined in Figure 1B. Representative CAG repeat distributions from liver of F1 progeny between CBy and other inbred strains of mice. The top, bottom and second panel show the controls CBy (stable), B6 (unstable), and CBy X B6 (intermediate) CAG profiles, respectively. Note: Western blot data comes from inbred mice. The higher levels of MSH3 in C3H and B6 are halved in the cross to CBy.

The higher levels of MSH3 in the B6 variant may be due to stabilizing amino acid sequences or alternatively, the lower levels of MSH3 in the CBy variant may be due to destabilizing amino acid sequences. Since the levels of MSH2 were consistent between the congenic and reciprocal congenics we presume that the contribution of MSH2 variants upon MSH3 levels is less than that of MSH3. Towards identifying *Msh3* gene polymorphisms that may affect MSH3 protein levels, we sequenced the *Msh3* gene from 12 other inbred mouse lines for promoter and exon 2, 3, 7, 8 and 10 variations (A, AKR, C3H, CBA, FVB, DBA/2, 129P2, 129S1, 129S2, 129S6, 129T2, & 129X1). These mouse lines contained variant amino acids similar to either CBy or B6 (Figure 4A, Table S2). We next assessed the MMR protein levels in various strains that harboured the B6 and CBy *Msh3* gene coding polymorphisms (Figure 4B). MSH3 expression varied between strains. MSH3 was barely detectable in CBy and was the highest in B6 and C3H/HEJ (Figure 4B). These MSH3 levels are similar to the lower and higher levels observed in our reciprocal congenic mice with the CBy- and B6-*Msh3* alleles, respectively. MSH3 is highest in B6 and C3H/HEJ mice, which share alleles in exon 3, exon 7 and exon 10 suggesting that these may contribute positively to MSH3 levels. MSH3 levels were intermediate in DBA/2J, CBA/J and 129/S1 (Figure 4B), and these all share the B6 variants at exon 10, which provides additional support for a stabilizing association of exon 10. This is further supported by the higher MSH3 expression in DBA/2 than CBy since DBA/2 differs from CBy by two polymorphisms in exon 10 (Figure 4A). Our results indicate that polymorphisms within exon 3, exon 7 and exon 10 may modulate the level of MSH3 protein in mouse tissue.

In further support for the CAG repeat-stabilizing effect of the CBy *Msh3* variant, we crossed the CBy.Cg-R6/1 mice to the above noted 12 strains of mice including B6, which contained different *Msh3* gene variants (Figure 4A, Table S2). All F1 mice regained an intermediate level of CAG instability in their liver and/or striatum, which is consistent with this set of *Msh3* variants being the source of altered CAG/CTG instability (Figure 4C). Notably, in 3 independent crosses of CBy.Cg-R6/1 to C3H/HeJ, which showed the highest expression of MSH3, all F1 mouse livers showed the same pattern of CAG instability with a broad distribution of expanded alleles extending to as many as +12 repeats. This dosage effect is consistent with a dominant effect of MSH3 levels upon CAG instability. Further support for a MSH3 dosage effect is the near complete absence of MSH3 protein in either tail or heart of either CBy or B6 mice with the exception of tail tissue of 4 week-old mice. This expression profile correlates with the relatively stable repeat tract observed in these tissues, regardless of mouse strain (Figure S5, see also Figure 1). These findings are consistent with a direct association of tissue-specific MSH3 levels with levels of tissue-specific CAG stability.

### T321I MSH3 variant is highly conserved and may destabilize MSH3 protein

**Figure 5:**
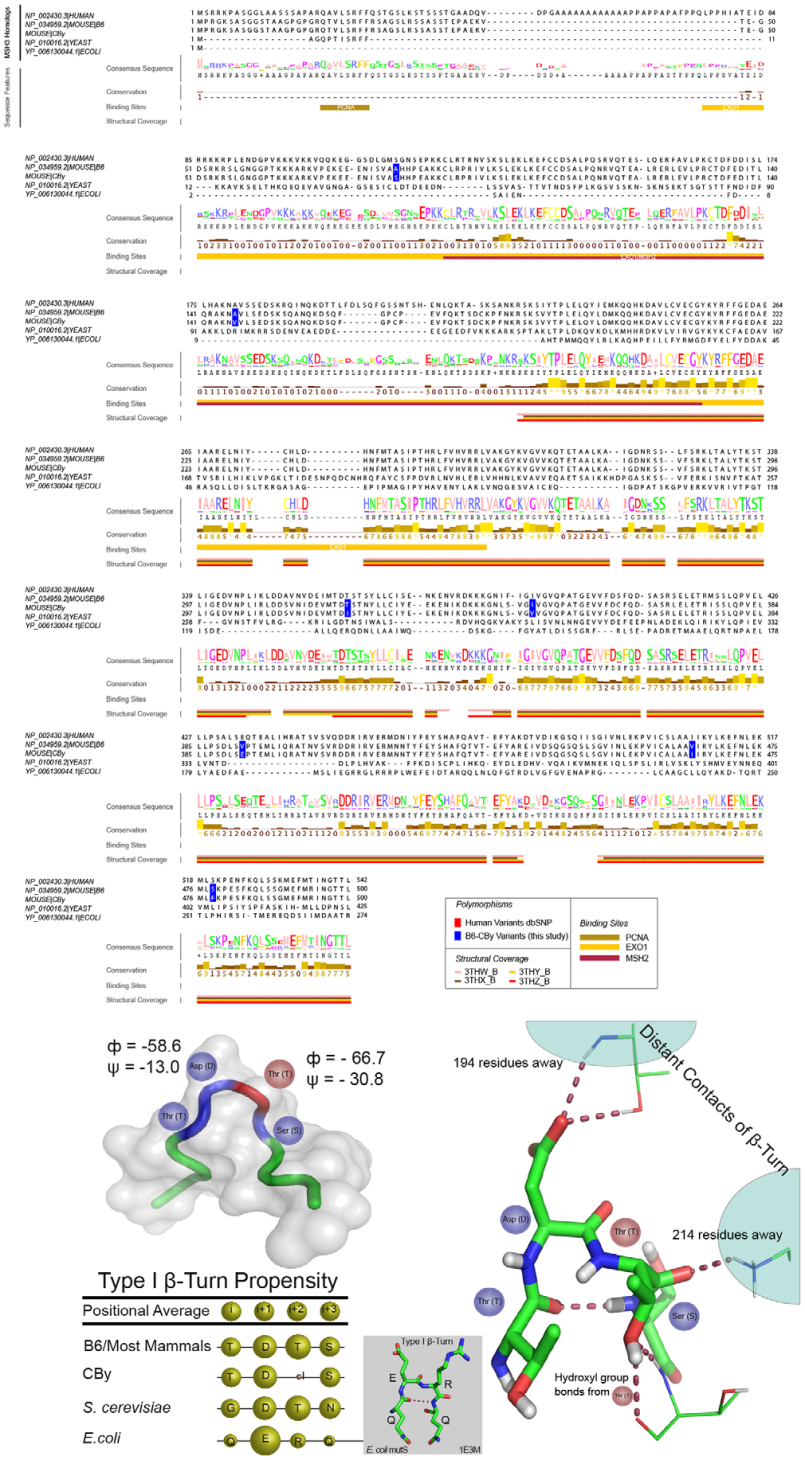
Structural and sequence analysis of MSH3. A: Multiple sequence alignment of MSH3. Jalview created visualization [120] using the first 500 amino acids of the mouse B6 MSH3 (NP_034959.2). Conservation values and consensus sequence are based on alignment of *S. cerevisiae* Msh3p, *E. coli* MutS and 17 mammalian MSH3 homologs; values range from 0–9, where 0 is lowest and 9 is the highest. Protein interacting domains indicated pertain to those regions of the human MSH3 protein. This panel only shows an abbreviated set of the species of MSH3 sequence, the full set analysed is shown in Figure S5. B: MSH3 variant within β-turn. The T321I variant occurs within a Type I β-turn, as determined by specific backbone turn angles [117], [118] from the human MSH3 structure (3THW_B). Top left: hMSH3 tube diagram of Cα atoms of β-turn (blue), *i*+2 (T) residue (red) and additional three residues on N- and C-terminal ends (green). Bottom left table shows the β-turn propensity is relatively strong throughout MutS/MSH3 homologs, while the CBy variant (Isoleucine at *i+2* position) is extremely disfavored (table bottom left) [69]. Right: Ball and stick diagram of contact sites of Asp (D) and Thr (T) residues in β-turn with residues 194 and 214 respectively. Line diagram of Thr (T) hydroxyl group contact with neighbouring Threonine residue at position 365. The absence of the Threonine hydroxyl group may be important to stabilizing the β-turn itself, and/or may change the conformation of the turn, potentially disrupting distant contacts important for proper protein folding. MSH3 visualizations created using PyMol (PyMOL Molecular Graphics System, Version 1.2r3pre, Schrödinger, LLC).

In order to uncover potential amino acid changes, which could be contributing to loss of MSH3 protein expression in the CBy variants, we have examined both sequence and structural features of MSH3 homologs. Sequence alignment has revealed that most of the B6-CBy variants are well conserved, but occur where amino acid changes are not predicted to have physiochemical consequences, or occur within poorly conserved regions, suggesting those regions minimally contribute to structure/function of the protein (Figure 5A). One exception is the T321I variant, which is conserved in 16/17 of the mammalian homologs and yeast. Further, in this one exception (in both giant panda and yeast), the Threonine is replaced by physiochemically-similar Serine, so that a hydroxyl group at this position is observed to be highly conserved (Figure 5A and Figure S6). Importantly, the T321 variant occurs within a Type I β-Turn (Figure 5B), where Isoleucine is extremely unfavoured (Figure 5B) [69]. Despite the large evolutionary distance, a Type I β-Turn also occurs in *E. coli* MutS (Figure 5B) [70], suggesting the importance of this region to overall function. β-Turns are thought to be crucial to the protein folding process [71], [72], where they may direct nucleation of secondary structure elements towards hydrophobic collapse [73]. The change of Threonine to disfavoured Isoleucine at the ‘i+2’ site within the turn of MSH3 may disrupt the β-Turn, representing a significant barrier to protein folding, potentially leading to proteolysis. The full effect of the T321I change upon MSH3 protein stability may require some of the other amino acid changes, which will require experimental assessment.

## Discussion

Trinucleotide repeat instability is governed by *cis*-elements and *trans*-acting modifiers. Repeat length, sequence of repeat, purity of the repeat, genomic context and DNA metabolizing proteins can contribute to patterns of repeat instability in mouse models of trinucleotide repeat diseases such as DM1, HD and SCA7 [19]. Since the R6/1 transgene is common to each of the mouse lines described herein, the variable levels of CAG instability between strains are unlikely to be the result of a *cis*-element, and most likely result from the different *Msh3* gene variants. Further support for a *trans*-factor as the source of the variable CAG stability is that B6, FVB, and 129 mouse strains did not influence *HTT* mRNA levels for either knock-in or YAC HD mice [59] thereby arguing against a role for transcription as an in *cis* source for the inter-strain variations of instability. To date there has been no report of a naturally occurring mouse strain-specific factor that modifies repeat instability.

In HD and DM1 mice, engineered null alleles of *Msh2* and *Msh3* were identified as the strongest modifiers of trinucleotide repeat instability suggesting an important role of MutSβ in trinucleotide repeat instability [9], [37]. MMR deficiencies stabilized CAG/CTG repeat tracts from spontaneous expansions in two different kinds of HD mice [13], [27], [36], [46] and three different DM1 mouse models [16], [26], [28], [34], [35], [74]. These results indicate that the effects of MMR proteins on CAG/CTG instability are independent of *cis*-elements and sequence context. We observed two distinct patterns of somatic CAG instability in two different *M.musculus* backgrounds, CBy and B6 and sequenced the *Msh3* gene in those strains and found seven polymorphisms in exons 2, 3, 7, 8 and 10, which differ between the strains. Thus, the differences in CAG/CTG instability between the two strains may be modulated by these *Msh3* polymorphisms. By generating reciprocal congenic mice for the *Msh3* gene, we demonstrated that CAG/CTG repeat instability appears to be modulated by *Msh3* variants, where expansion levels are the highest in liver and striatum of mice homozygous for the B6 *Msh3* gene. Mice homozygous for the CBy *Msh3* gene show an absence of CAG instability. We also showed that MSH3 protein expression depends upon the *Msh3* gene variant, independent of genetic background outside the *Msh3* locus: The B6 MSH3 protein variant was expressed at high levels, whereas the CBy MSH3 variant was expressed at nearly undetectable levels. The protein expression patterns of MSH3 correlated positively with the level of somatic CAG expansions. The loss of one B6 *Msh3* allele in mice heterozygous for both variants was sufficient to decrease CTG/CAG instability; consistent with results which shows that MSH3 protein levels are a limiting factor in CAG/CTG repeat expansions in DM1 and HD mouse models, where MSH3/null mice have less expansions than MSH3/MSH3 mice, but more than null/null mice [16], [27], [35]. Interestingly, the loss of one *Msh3* allele (*Msh3*/null) was more dramatic than the loss of one *Msh2* allele [13], [27], [35], suggesting that CAG instability may be exquisitely sensitive to MSH3 levels. In a repair assay, the levels of human MSH3 protein altered the ability to repair slipped-DNAs formed by CAG/CTG repeats [42]. The distinct levels of MSH3 protein between B6 and CBy strains are unlikely to be due to varying transcription levels, as we detected similar levels of the DHFR protein between strains, whose transcript is driven from the same divergently transcribed promoter as the *Msh3* gene [66]–[68]. Furthermore, considerable evidence indicates that the levels of MMR transcripts is not always reflective of MMR protein levels [48]. The stability of MSH3 and MSH6 proteins is dependent on the ability of these proteins to form heterodimeric complexes [48]; in mice the genetic absence of *Msh2* led to undetectable levels of MSH3 protein [45]. However, the levels of MSH2 protein did not vary between the B6 and CBy strain (Figure 3), and MSH3 protein levels (low or high) persisted in the reciprocal congenic mice; arguing against variations of MSH3 levels by either strain-specific MSH2 expression levels or MSH2 variants.

Polymorphisms in the MSH3 coding region may alter the stability of the MSH3 protein directly or by altering its interaction with MSH2 [47], [48]. In particular, although our homology modeling results did not offer insight into which variants resided in regions critical to overall protein structure nor did the polymorphism reside in known protein-binding domains (Figure 5A and Figure S6), the highly conserved T321I variant occurs within a Type I β-Turn which could be critical for protein folding [71], [72]. Changes in β-Turn sequences modulate protein stability [71], [72], [75], [76], where unfavourable sequence changes can dramatically decrease protein folding rate [77], [78] and in some cases completely ablate protein expression [79]. In addition to potential changes brought by the T321I variant, the CBy strain gains a potential serine phosphosite at amino acid 79 as experimentally determined in the homologous human MSH3 protein (site 116 in hMSH3) [80], which could impact overall protein conformation, its protein-binding capacity and stability [81]. While the actual contribution of any of the MSH3 amino acid variants, alone or coincident with the others, will require experimental support, together our findings support an effect upon protein stability.

Here we have shown that naturally occurring genetic variation in an MMR gene, like engineered genetic deficiencies of MMR genes, can lead to changes in the direction and pattern of CAG/CTG repeat instability. A loss of *Msh2* and *Msh3* have led to both a loss of expansions and increased CAG/CTG contractions; suggesting that MMR proteins may both drive expansions as well as protect against contractions [16], [26], [27], [34]–[36], [49]. The pattern of CAG instability is also affected by MMR genes – possibly reflected by changes in the number of repeat units involved in a mutagenic event. It is possible that there are two different mechanisms involved in large expansions; the accumulation of many short (single-repeat) length changes per mutagenic event; or salutatory large (many-repeat) jumps per mutagenic event. *In vivo* evidence suggesting the existence of two distinct mechanisms was the observation of bimodal distribution of repeat length in certain tissues of HD and DM1 mice [13], [27], which is also evident in some patient tissues and may be due to cell lineage-specific instabilities [4], [82]–[84]. This bimodal distribution of repeat lengths was only observed herein with homozygosity for the B6 *Msh3* gene (Figure 1 and Figure 2). Recent modeling studies of HD mice suggest the involvement of distinct short and large mutagenic events [25]. Similarly, it was reported that two distinct modes of repeat instability occur at dinucleotide repeats in MMR-defective (hMSH2, hMLH1, hPMS2) deficient tumours of humans but not mice, those with changes of < or = 6 repeats and those with changes of >8 repeats [85]–[87]. In cultured cells of patients suffering a CAG/CTG disease and certain tissues the mutation events appear to be short increments, of 1 to 3 CTG/CAG units per mutation event [88], similar to that occurring at other simple repeats such as (CA)n and (A)n [85], [89], [90]. Interestingly, the bimodal distribution of CAG expansions was lost in mice harbouring a CBy *Msh3* gene, which might suggest that MSH3 is involved in larger repeat expansions. However, the requirement of hMutSβ for the repair of short slip-outs of a single repeat unit, but not of longer slip-outs (>3 units), strongly supports the concept that the MSH3-sensitive expansions we observe in the mice are in fact the accumulation of many single-repeat expansion events [42].

An effect of mouse genetic backgrounds on the dynamics of CAG/CTG expansions was suggested in HD and DM1 mouse models, but a candidate for the source for the variation was not suggested [16], [59]. van den Broek *et al*., (2002) showed the greatest CTG instability when present on the C3H background, while Lloret et al., (2006) observed the highest CAG instability on a B6 background and low levels of instability in the 129Sv background [16], [59]. These observations with independent transgenic mice showing the highest repeat instability in mice with the B6 *Msh3* gene (C3H and B6) and lower instability in mice with the CBy *Msh3* gene (129) are consistent with our findings that the B6 MSH3 variant is a major driver of CAG expansions, and is also consistent with the high levels of MSH3 protein in B6 and C3H mice. It is unclear why these *Msh3* polymorphisms, which appear to affect MSH3 protein levels, exist in the various mouse lineages. We propose that differences in the MSH3 protein between mouse strains may provide a molecular explanation for some of the strain-specific differences observed in somatic CAG instability seen by other labs [16], [59].

DNA polymorphisms in other DNA metabolizing proteins might affect CAG/CTG instability patterns, and such family-specific instability modifiers have been suggested to exist in HD families [61]. Many *trans*-factors have been considered for their role in CAG/CTG instability, and few have been assessed for the possible contribution of their polymorphic variants. Neither human FEN1 mutants nor its polymorphic variants were linked to CAG instability in HD patients [91]. OGG1 has been reported to play a partial role in CAG instability in R6/1 mice [33]. Huntington's subjects having the Cys326-OGG1 allele were reported to have increased HD CAG tract lengths and significantly earlier disease onset than HD individuals with the Ser326 variant [92]. However, this association was not observed in a study using a larger sample size [93]. Our recent observation that the human mismatch repair protein MLH1 is required to repair short CTG slip-outs and arrested on clustered slip-outs, might suggest that MLH1 is involved in CAG/CTG expansions, and MLH1 variants may have differential effects [94]. Polymorphisms in human *MSH2* have been identified in patients with hereditary non-polyposis colorectal cancer that are thought to inactivate the function of the MSH2–MSH3 complex but not the MSH2–MSH6 complex; leading to altered frameshift mutations in yeast [95], [96]. Polymorphic variants of *hMSH3* have been significantly linked to cancer and radiation sensitivity [97]–[100]. However, in no case has there been any demonstration of altered genetic variation with a particular *hMSH3* variant, nor any direct link of an hMSH3 variant with a mutagenic outcome.

Might polymorphisms of *Msh3* affect the instability of other repeat sequences? Mismatch repair proteins act in distinct manners upon the instability/stability of different repeats. A loss of *Msh3* can lead to varying levels of changes of single repeat units (predominantly losses) at mono- di-, tri- and tetranucleotide repeat tracts [101] and references therein). The role of mismatch repair proteins in the instability of expanded repeat sequences including the FRDA disease-associated GAA tracts, the murine Ms6-hm (also known as Pc-1) (CAGGG)n and Hm-2 (GGCA)n repeats can vary widely from their effects upon CAG/CTG repeats [102]–[106]. Together these findings support the contention that the role of MMR can vary dramatically across different repeat sequences. Thus, the effect MMR gene polymorphisms on different repeat sequences will need to be determined for each sequence. However, since the *Msh3* variants appeared to have similar effects upon CAG/CTG instability in various transgenic contexts, the effect of MMR gene polymorphisms may be similar for each of the 14 different CAG/CTG disease loci including HD, DM1, SCA7, and others.

Our data provide the first evidence that *Msh3* polymorphic variants associate with levels of CAG/CTG trinucleotide instability in HD mice. This discovery may lead to the identification of human polymorphic variants that could explain the extreme variability of CAG/CTG instability observed in HD and DM1 patients. Since somatic repeat expansions through an individual's life may contribute to disease severity and progression, factors that affect this could have clinical relevance [1], [7], [8], [9]. Unknown genetic factors modify the onset and severity of disease in HD families and HD mice [60], [107]–[109]. Un-explored variations in the levels of somatic CAG instability between HD families or individuals may be the source for these clinical variations. Polymorphic variants in DNA repair genes that lead to enhanced somatic CAG/CTG expansions could ultimately lead to increased disease progression and severity. Similarly, variants that lead to reduced somatic expansions could be less deleterious. Identification of such variants in individuals affected with any one of the 14 CAG/CTG diseases may have prognostic implications. Furthermore, attempts to modulate MMR to modulate CAG/CTG-repeat associated diseases [9] would be wise to consider any particular variants of MMR proteins that may differentially affect instability levels.

## Materials and Methods

### Mouse breeding

This study was performed in strict accordance with the recommendations in the Guide for the Care and Use of Laboratory Animals of the National Institutes of Health. The protocol was approved by the Institutional Animal Care and Use Committee of the Wadsworth Center (Public Health Service Animal Welfare Assurance Number A3183-01).

Creation of R6/1 congenic lines of mice (CBy.Cg-R6/1 and B6.CBy-R6/1): male B6CBA-Tg(HDexon1)61Gpb mice originally purchased from Jackson Labs (Bar Harbor, ME), were bred to recipient strain CBy and B6 females to create F1 progeny. Male R6/1 transgenic F1 progeny were then backcrossed as appropriate to CBy or B6 females. Male R6/1 progeny from this cross (N2 generation) were selected for backcross to the appropriate recipient strain until 10 backcrosses had occurred (N10 generation). All R6/1 mice were genotyped from tail tip biopsy taken at weaning, using primers 1594 (CCGCTCAGGTTCTGCTTTTA) and 1596 (TGGAAGGACTTGAGGGACTC). PCR conditions were initial denaturation for 2 min at 95°C, followed by 30 cycles (94°C - 30 sec., 54°C - 45 sec., 72°C – 1 min), followed by 10 min at 72°C. Qiagen Taq polymerase (cat #201225) was used as recommended by manufacturer.

Creation of *Msh3* reciprocal congenic lines of mice (CBy.B6-msh3 and B6.CBy-msh3): similar to creation of the R6/1 congenic mice, B6 and CBy inbred mice were intercrossed to produce F1 progeny. F1 progeny were backcrossed to recipient B6 and CBy inbred lines until attainment of the N10 generation. Starting with N2 progeny, mice were genotyped with markers that flanked the *Msh3* gene. D13Mit159 (Forward: CCCATTGTCCCTGTTCAGAT, Reverse: AAACCCACCATGAATTAAATGC, position: Chr13:92953376–92953513 bp) and D13Mit147 (Forward: CATCCAGGAAGGCAATAAGG, Reverse: CAAATGCACAGTGCCGAG, position: Chr13:98359080–98359187 bp), were used for genotyping the locus. The *Msh3* gene is located at Chr13:93121206–93121391 bp. Animals heterozygous for both markers at each generation were selected as breeders. Qiagen *Taq* polymerase (cat #201225) was used as recommended by the manufacturer.

Creation of double congenic lines: CBy.B6-msh3^B6/B6^ females were crossed to CBy.Cg-R6/1 males, and progeny were genotyped at both D13Mit markers and for the R6/1 transgene. Females heterozygous at the *Msh3* locus and males heterozygous at the *Msh3* locus and carrying the R6/1 transgene were selected for mating. Female progeny of this mating typing either homozygous B6 or CBy at the *Msh3* locus were mated with a male B6 or CBy *Msh3* homozygotes who also carried the R6/1 transgene to establish CBy.B6-msh3^B6/B6^ R6/1 and to derive a control CBy.Cg-R6/1 (homozygous CBy at the *Msh3* locus) lines. The same procedure was used to create B6.CBy-msh3^CBy/CBy^ R6/1 and control B6.Cg-R6/1 lines.

### Genome-wide SNP genotyping

The efficacy of our congenic and reciprocal congenic mice was assessed by SNP genotyping with a medium-density SNP array (Mouse MD Linkage panel #GT-18–131, Illumina, San Diego, CA) on DNA samples isolated from mouse tail clips using the GoldenGate Genotyping Assay according to the manufacturer's protocol. This allowed us to precisely map the recombination boundaries and test for contaminating regions. The microarray detects 1449 loci where 796 are informative between C57BL/6 and BALB/cBy, excluding those on the X chromosome. These loci span the entire mouse genome with approximately three SNPs per 5 Mb intervals. Briefly, 250 ng of DNA (5 uL at 50 ng/uL) was hybridized to locus-specific oligonucleotides, extended, ligated and amplified before hybridizing to universal 1,536-plex 12-sample BeadChip microarrays. The arrays were then scanned with default settings using the Illumina iScan. Analysis and intra-chip normalization of resulting image files was performed using Illumina's GenomeStudio Genotyping Module software v.2011 with default parameters. Genotype calls were generated by clustering project samples with a manual review of each SNP plot. The identified contaminating SNPs were visualized by ideogram using the Ideographica web-based software [110].

### CAG repeat analysis

Genescan Analysis: purified gDNA from tissue and 10 ng was amplified using primers HDSizeF (6FAM-ATGAAGGCCTTCGAGTCCCTCAAGTCCTTC) and HDSizeR (CGGCGGTGGCGGCTGTTG). PCR conditions were initial denaturation for 3 minutes at 94°C, followed by 30 cycles (94°C (30 s.), 58°C (1 min.), 72°C (1 min), followed by 10 minutes at 72°C. Qiagen *Taq* polymerase (cat #201225) was used as recommended by manufacturer. Products were processed in the Applied Genomics Technologies Core at the Wadsworth Center on an ABI3730, and analysed using PeakScanner Software (Applied Biosystems).

Small pool-PCR: DNA from testis was extracted by phenol-chloroform and sperm was extracted as described [26],[34],[74]. SP-PCR was performed as described [111]. DNA samples were digested with *Hin*dIII and SP-PCR was performed with MS-1F (GCCCAGAGCCCCATTCATT) and MS-1R (GGCTACGGCGGGGATGGCGG) primers. The DNA was denatured by heating to 94°C (5 min.) and amplified through 30 cycles of 94°C (1 min.), 62°C (1 min.) and 72°C (1 min.) with a chase of 10 minutes at 72°C. The products of the PCR were resolved by electrophoresis on 40 cm long 1.5% agarose gels in 0.5× TBE at 180 V for 18 hours. The products were then transferred to nylon membrane by Southern blotting and detected by hybridization using a radiolabelled CAG repeat containing probe.

To assess degrees of instability we used the following criteria: The presence of instability was evidence by multiple PCR products with varying lengths of repeats. The degree of instability between different tissues was assessed based upon the size range and the relative amount of the expanded product was different from the major sized product in the stable tissues (presumed as the progenitor allele; most studies indicate the tail as representative of the progenitor allele). The degree of instability for the same tissue between different mouse lineages was assessed in a similar manner. The above were done on an age-matched basis. Relative between age-matched and tissue matched mice, an assessment of the size range and the intensity of the fragments as previously outlined [13], [17], [18], [29], [47], [111].

### LOD score calculation

LOD score was calculated using numbers of mice from the F1 intercross = 81. The F1 parents were all by definition heterozygous, therefore the 81 mice derive from 2 * 81 = 162 informative meiosis. Assuming the phenotype is mediated by two linked semi-dominant alleles, then there are no recombinants observed (i.e., all the mice have the phenotype expected consistent with their genotype). Thus the number of recombinants = 0 and the number of non-recombinants = 162. The odds of getting this outcome assuming linkage and 0% recombination (θ = 0%) = 1. The odds of getting this outcome if the loci were not linked = 0.5162 = 1.7×10–49. The LOD score is then calculated as the log of the odds of observing this pattern assuming no linkage/assuming linkage = log (1/1.7×10–49) = 48.8 (θ = 0)

### Protein sample preparation and Western blotting

Tissues were collected from 4 and 16-week-old-mice with different *Msh3* genotypes. Protein extractions and Western blotting were performed as described in Tomé *et al*., (2013) [45]. Briefly, proteins were extracted by mechanical homogenisation in lysis buffer (0.125 M, Tris-HCl (pH 6.8), 4% SDS, 10% glycerol) containing protease inhibitor cocktail (Roche, complete Mini 7× cat. no. 04 693 124 001). Protein concentration was determined using the Pierce BCA protein assay kit (cat. no. 23225). Proteins (40 µg) were denatured for 5 minutes at 95°C in loading buffer and 10% β-mercaptoethanol added extemporaneously, resolved by electrophoresis on an 8% (MMR proteins and actin expression) and 12% (DHFR expression) SDS-PAGE. Membranes were blocked for one hour at room temperature in 5% (m/v) dried milk for MMR antibodies incubation and 10% (m/v) dried milk for DHFR incubation in TBST pH 7.5, then incubated overnight at 4°C in antibodies anti-MSH2 (Ab-2) mouse mAb (FE11) (Calbiochem, Ab-2; cat. no. NA27, 1∶200), mouse anti-MSH6 (BD Laboratories, cat. no. 610918, 1∶200), monoclonal mouse anti-MSH3 (2F11 and 5A5 clones, gifts from Glen Morris and Ian Holt, at 1∶750) [65], Rabbit anti-DHFR [112] (gift from Joseph R. Bertino, 1∶500) and mouse anti-Actin Ab-5 (BD laboratories, cat.no. 612656, 1∶500). The anti-MSH3 antibodies 2F11 and 5A5 recognize epitopes in exons 1 and 4, respectively [65]. The membranes were incubated for 1 h in mouse secondary antibody (sheep anti-mouse-HRP, cat. no. 515-035-062, 1∶5,000) at room temperature for MSH2, MSH3, MSH6 and for actin and Rabbit secondary antibody (Abcam, Rabbit polyclonal to goat IgG H and L, HRP, cat. no. ab6741) for DHFR. Antibody binding was visualized using ECL plus Western blotting detection system (Amersham, cat. no. RPN2132).

Spleen extracts were prepared from fresh spleens placed in 3 ml cold PBS with 2% FBS to rinse away excess erythrocytes. Spleens were passed through nylon mesh filter (BD Falcon cell strainer, 40 mm, REF352340) containing fresh cold PBS with 2% FBS and 2 mM EDTA on ice, then centrifuged at 1200 rpm at 4°C for 5 minutes, and processed as outlined [42].

#### Sequence alignment

Mammalian homologs of MSH3 were obtained using 5-iteration PSI-BLAST [113] with E-value set to 1e-05 against mouse MSH3 (NP_304959.2). Results were filtered to exclude MSH6 proteins and partial or low quality proteins, leaving 17 mammalian MSH3 homologs (including mouse). Mammalian sequences were chosen to provide consistent dataset where structural features of MSH3 are likely conserved. *Saccharomyces cerevisiae* 288c Msh3p, *Escherichia coli* str. K-12 substr. MG1655 MutS sequences and the 17 mammalian homologs were aligned using MAFFT with default settings [114]. Mammalian homologs were aligned using MAFFT [114].

#### Structure modeling

The hMHS3 structure 3THW_B from the Protein Data Bank (PDB) [115], offered the greatest coverage of the MSH3 sequence and has an extremely high percent identity to mouse MSH3 (87.1%), strongly suggesting similar structures for both mouse and human MSH3. Efficient side-chain packing of 3THW_B was achieved using SCWLR4 software [116] and the DSSP program [117] was used to assign secondary structure and phi/psi bond angles. β-Turn type was determined based on [118] and confirmed using PROMOTIF [69], [119]. 3THW_B structures were visualized using PyMol. Protein bonds were assigned with PyMol and distant contacts confirmed using an in-house Python script.

## Supporting Information

Figure S1 Genome-wide SNP analysis to localize the contaminating regions in congenic and reciprocal congenic mice. A) To determine the locations of contaminating donor genome in the *HTT* R6/1 transgene congenics, genome-wide SNP analysis of congenic strains and their parental strains was performed using the Illumina Mouse Medium Density Linkage Panel. The identified contaminating SNPs were visualized by ideogram using the Ideographica web-based software [110]. The *HTT* R6/1 transgene (red box) and the *Msh3* gene (blue box) location is noted on chromosome 3 and chromosome 13 respectively. Dark green dots represents contamination in B6.Cg, R6/1 congenic strain. B) To determine the locations of contaminating donor genome in the *Msh3* locus reciprocal congenic mice, genome-wide SNP analysis of reciprocal congenic strains and their parent congenics was performed using the Illumina Mouse Medium Density Linkage Panel. The identified contaminating SNPs were visualized by ideogram using the Ideographica web-based software [110]. Regions of CBy contamination in the B6.CBy-msh3 strain (dark green dots); B6 contamination in the CBy.B6-msh3 strain (light green dots) and areas of common contamination in both CBy.B6-msh3 and B6.CBy-msh3 (black dots) are shown. The *HTT* R6/1 transgene (red box) and *Msh3* gene (blue box) locations are noted on chromosome 3 and chromosome 13 respectively. For details see Table S1 and Figure S7.
(TIF)

Figure S2 CAG repeat stability in testes and germline. Representative SP-PCR analyses of CAG repeats in DNA molecules extracted from testes and sperm of 12- and 24-week-old transgenic mice of congenic or reciprocal congenic mice.
(TIF)

Figure S3 Western blot analysis of MSH3 protein level in different mouse tissues. MMR expression in spleen, thymus, cerebellum and cortex from 4 and 16 week-old mouse. Actin was used as a loading control. MSH3 2F11: 127 kD (dilution 1/750) and Actin: 42 kD (dilution 1/5000).
(TIF)

Figure S4 Western blot analysis of MSH3 protein level using two distinct antibodies to different MSH3 epitopes. Variable expression levels of MSH3 protein were detected using two independent monoclonal antibodies directed to different epitopes of MSH3. The anti-MSH3 antibodies 2F11 and 5A5 recognize epitopes in exons 1 and 4, respectively [65], neither of which have amino acid differences between B6 and CBy mice). Shown is the analysis of MSH3 from the testis of the indicated mice. The similar levels detected by the distinct antibodies reveals that, the MSH3 levels observed in tissues are independent of the binding site of the antibody on MSH3. Thus, regardless of genetic background, the level of MSH3 protein expression depended upon whether the mouse carried the B6 *Msh3* variant (high) or the CBy *Msh3* variant (low).
(TIF)

Figure S5 Western blot analysis of MSH3 protein level in heart and tail. MSH3 expression in tail and heart from 4 and 16 week-old mice. Western blot using only anti-MSH3 (Ab = 2F11) and actin antibodies in tail (left panel) and heart (right panel) from 4 and 16 week-old mice. Short exposure (top panel) and long exposures (bottom panel) are shown. MSH3 expression detected at low levels in tail of 4 week-old mice but not in 16 week-old mice. Undetectable levels of MSH3 in 4 and 16 week-old mice from heart tissue. MSH2 and MSH6 not detected in heart tissue of 4 and 16 week-old mice and low level detection of MSH2 in tail of 4 week-old (data not shown).
(TIF)

Figure S6 Full multiple sequence alignment of MSH3. Jalview created visualization of MSH3 alignment based on *S. cerevisiae* Msh3p, *E. coli* MutS and 17 mammalian MSH3 homologs. Conservation values and consensus sequence are based on all sequences excluding mouse CBy. Human polymorphisms (red block residue) do not map to mouse B6-CBy variants (blue block residues).
(TIF)

Figure S7 Contaminating genes flanking the *Msh3* gene in the reciprocal congenics. The contaminating regions linked to the *Msh3* gene in the reciprocal congenics. List representing *Msh3*-linked loci in the CBy.B6-*Msh3* R6/1 and B6.CBy-*Msh3* R6/1 reciprocal congenic mice. The contaminating regions linked to the *Msh3* gene in the reciprocal congenics contain a limited number of genes, none of which have an obvious or documented role in CAG repeat instability. The regions linked to the *Msh3* gene in the CBy.B6-Msh3 R6/1 reciprocal congenic mice span 43 Mbp and include 314 genes, of which 233 are protein-coding. In the B6.CBy-Msh3 R6/1 strain, the linked genes cover a region of approximately 22 Mbp, which lies within the 43 Mbp region of the CBy.B6-Msh3 R6/1 strain. A total of 151 genes are found within this region with 104-protein coding transcripts.
(TIF)

Table S1 SNP markers identified in the contaminating regions of both the congenic and reciprocal congenic mice listed with SNP marker name; chromosome number and position; R6/1 transgene and *Msh3* gene integration and allelic representation of donor strain. Contaminating SNPs are highlighted in red. All B6 alleles are indicated with a B and all CBy alleles indicated with an A.
(TIF)

Table S2 MSH3 coding polymorphisms in 14 different mouse strains. MSH3 protein polymorphisms from C57BL/6 (B6) and BALB/CBy (CBy) mice. SNPs were identified or confirmed to those in *dbSNP* by sequencing the *Msh3* gene, where similar amino acids were due to similar codons. In DBA/2J, exon 8, AA#392 was correctly identified to be T/Valine. For a given amino acid the same codon was used for the variants.
(TIF)
