# Supplementary figures and images for: MSH3 Polymorphisms and Protein Levels Affect CAG Repeat Instability in Huntington's Disease Mice
Source: PLoS Genet. 2013 Feb 28;9(2):e1003280. doi: 10.1371/journal.pgen.1003280 (PMC3585117; doi:10.1371/journal.pgen.1003280)

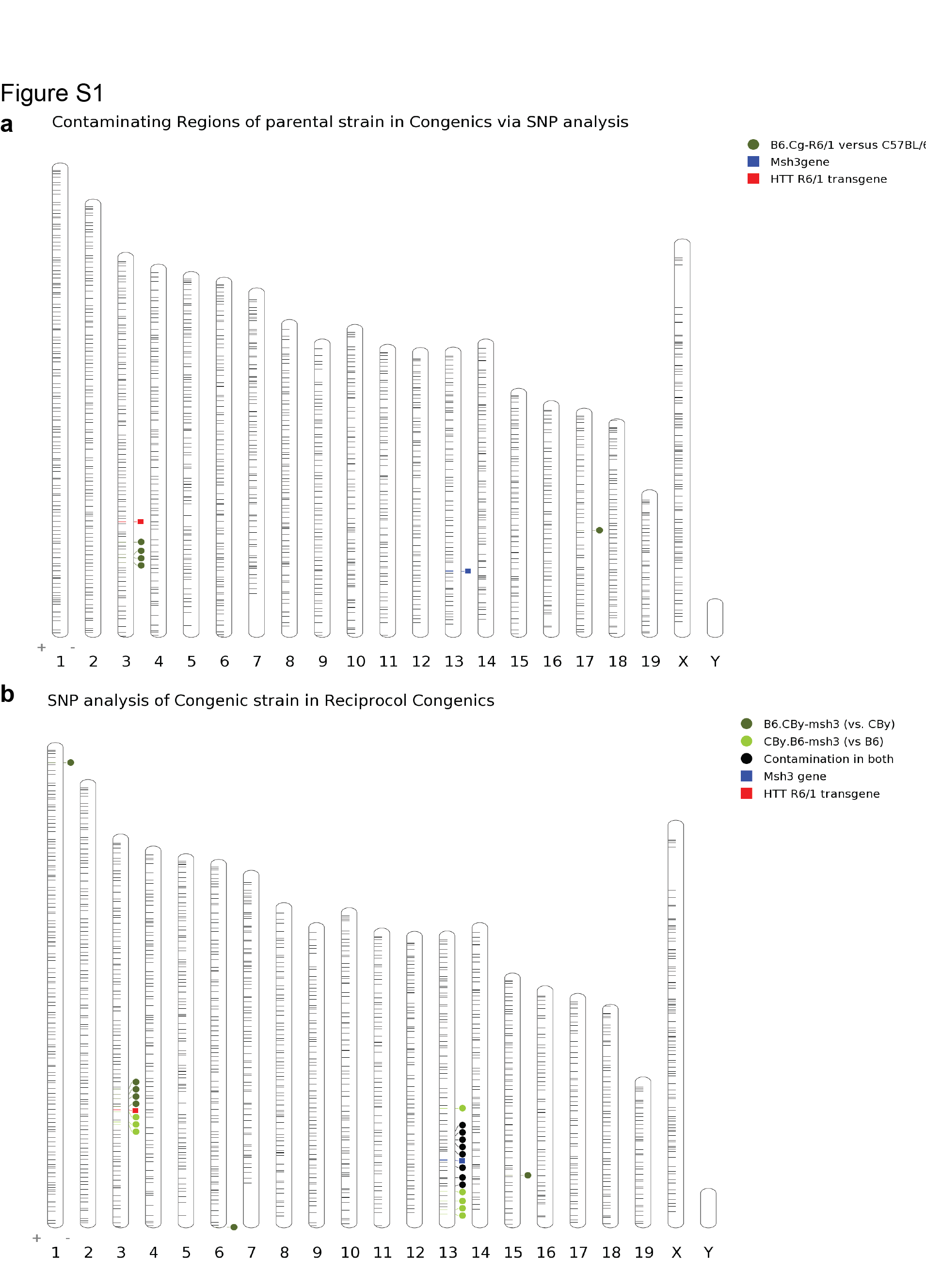

Supplement: Figure S1 — Genome-wide SNP analysis to localize the contaminating regions in congenic and reciprocal congenic mice. A) To determine the locations of contaminating donor genome in the HTT R6/1 transgene congenics, genome-wide SNP analysis of congenic strains and their parental strains was performed using the Illumina Mouse Medium Density Linkage Panel. The identified contaminating SNPs were visualized by ideogram using the Ideographica web-based software [110]. The HTT R6/1 transgene (red box) and the Msh3 gene (blue box) location is noted on chromosome 3 and chromosome 13 respectively. Dark green dots represents contamination in B6.Cg, R6/1 congenic strain. B) To determine the locations of contaminating donor genome in the Msh3 locus reciprocal congenic mice, genome-wide SNP analysis of reciprocal congenic strains and their parent congenics was performed using the Illumina Mouse Medium Density Linkage Panel. The identified contaminating SNPs were visualized by ideogram using the Ideographica web-based software [110]. Regions of CBy contamination in the B6.CBy-msh3 strain (dark green dots); B6 contamination in the CBy.B6-msh3 strain (light green dots) and areas of common contamination in both CBy.B6-msh3 and B6.CBy-msh3 (black dots) are shown. The HTT R6/1 transgene (red box) and Msh3 gene (blue box) locations are noted on chromosome 3 and chromosome 13 respectively. For details see Table S1 and Figure S7. (TIF) [file pgen.1003280.s001.tif]

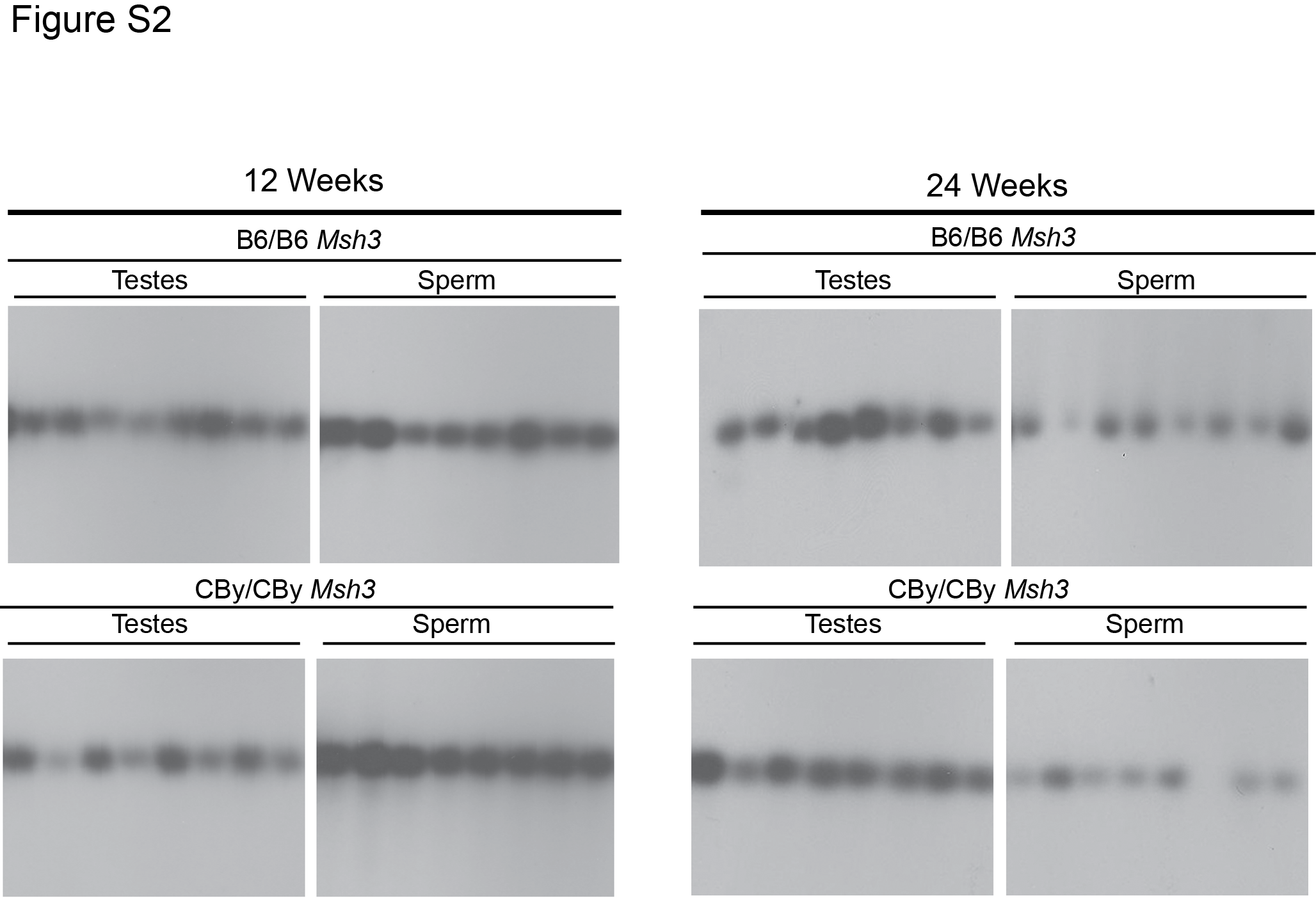

Supplement: Figure S2 — CAG repeat stability in testes and germline. Representative SP-PCR analyses of CAG repeats in DNA molecules extracted from testes and sperm of 12- and 24-week-old transgenic mice of congenic or reciprocal congenic mice. (TIF) [file pgen.1003280.s002.tif]

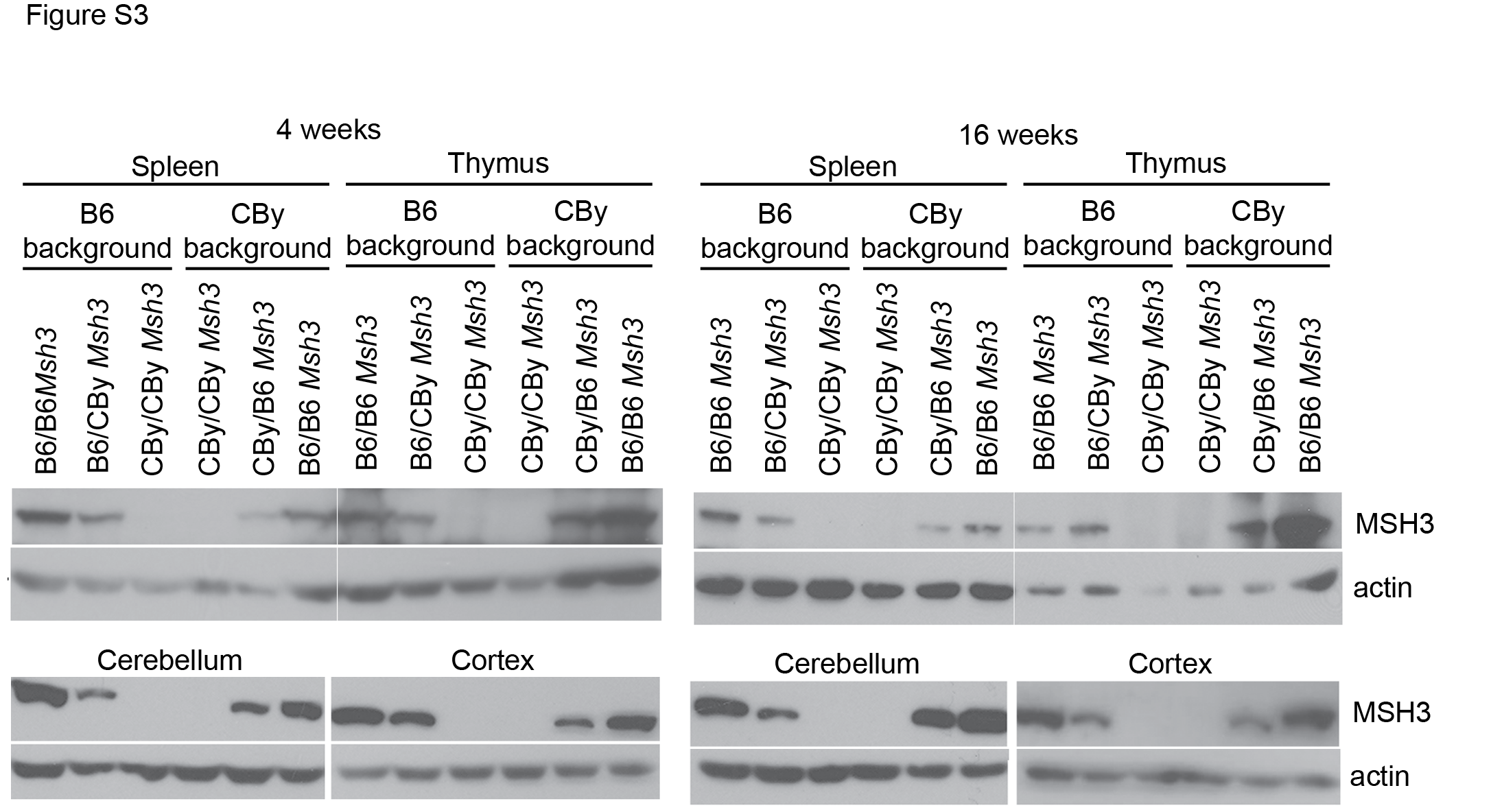

Supplement: Figure S3 — Western blot analysis of MSH3 protein level in different mouse tissues. MMR expression in spleen, thymus, cerebellum and cortex from 4 and 16 week-old mouse. Actin was used as a loading control. MSH3 2F11: 127 kD (dilution 1/750) and Actin: 42 kD (dilution 1/5000). (TIF) [file pgen.1003280.s003.tif]

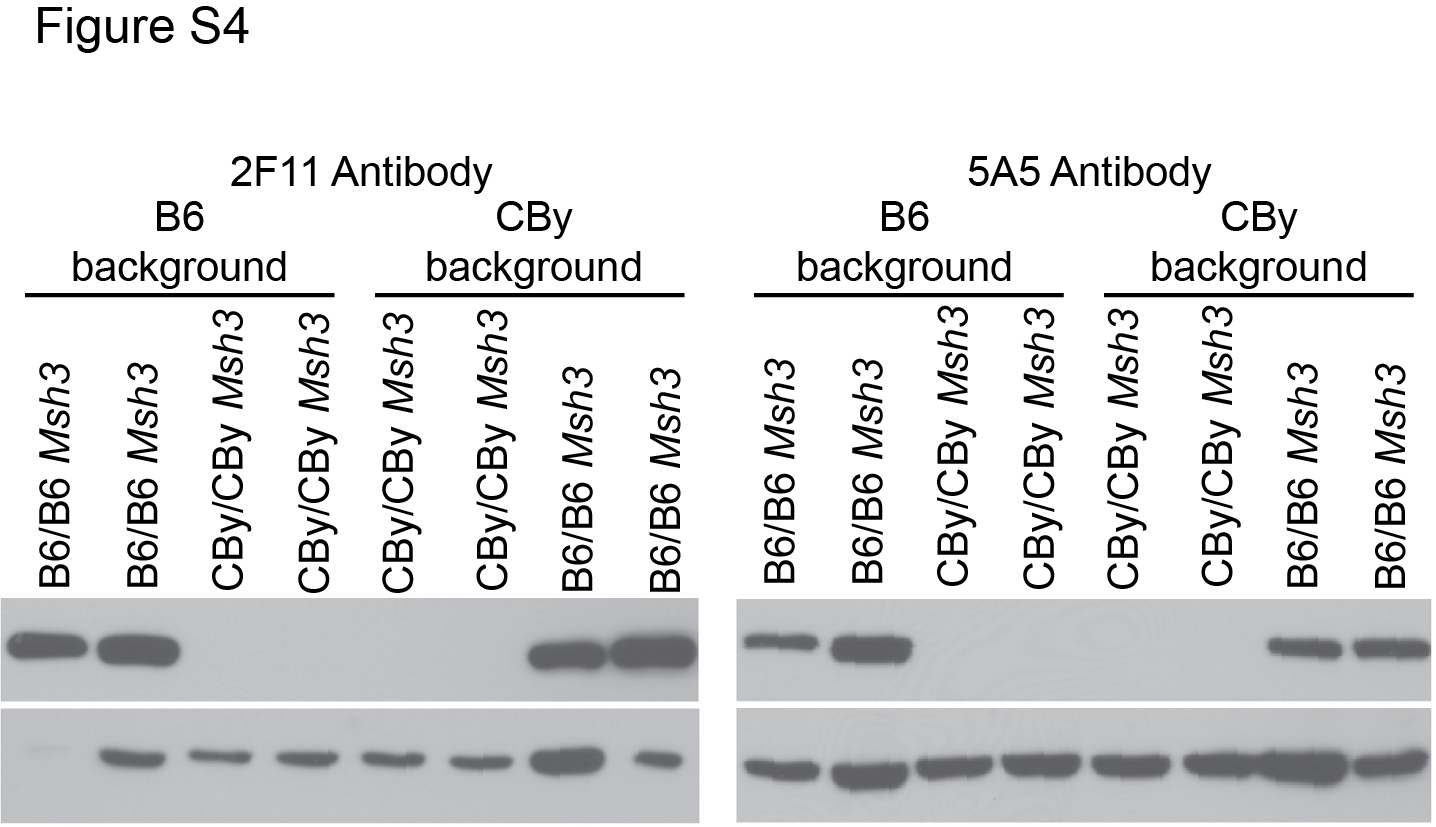

Supplement: Figure S4 — Western blot analysis of MSH3 protein level using two distinct antibodies to different MSH3 epitopes. Variable expression levels of MSH3 protein were detected using two independent monoclonal antibodies directed to different epitopes of MSH3. The anti-MSH3 antibodies 2F11 and 5A5 recognize epitopes in exons 1 and 4, respectively [65], neither of which have amino acid differences between B6 and CBy mice). Shown is the analysis of MSH3 from the testis of the indicated mice. The similar levels detected by the distinct antibodies reveals that, the MSH3 levels observed in tissues are independent of the binding site of the antibody on MSH3. Thus, regardless of genetic background, the level of MSH3 protein expression depended upon whether the mouse carried the B6 Msh3 variant (high) or the CBy Msh3 variant (low). (TIF) [file pgen.1003280.s004.tif]

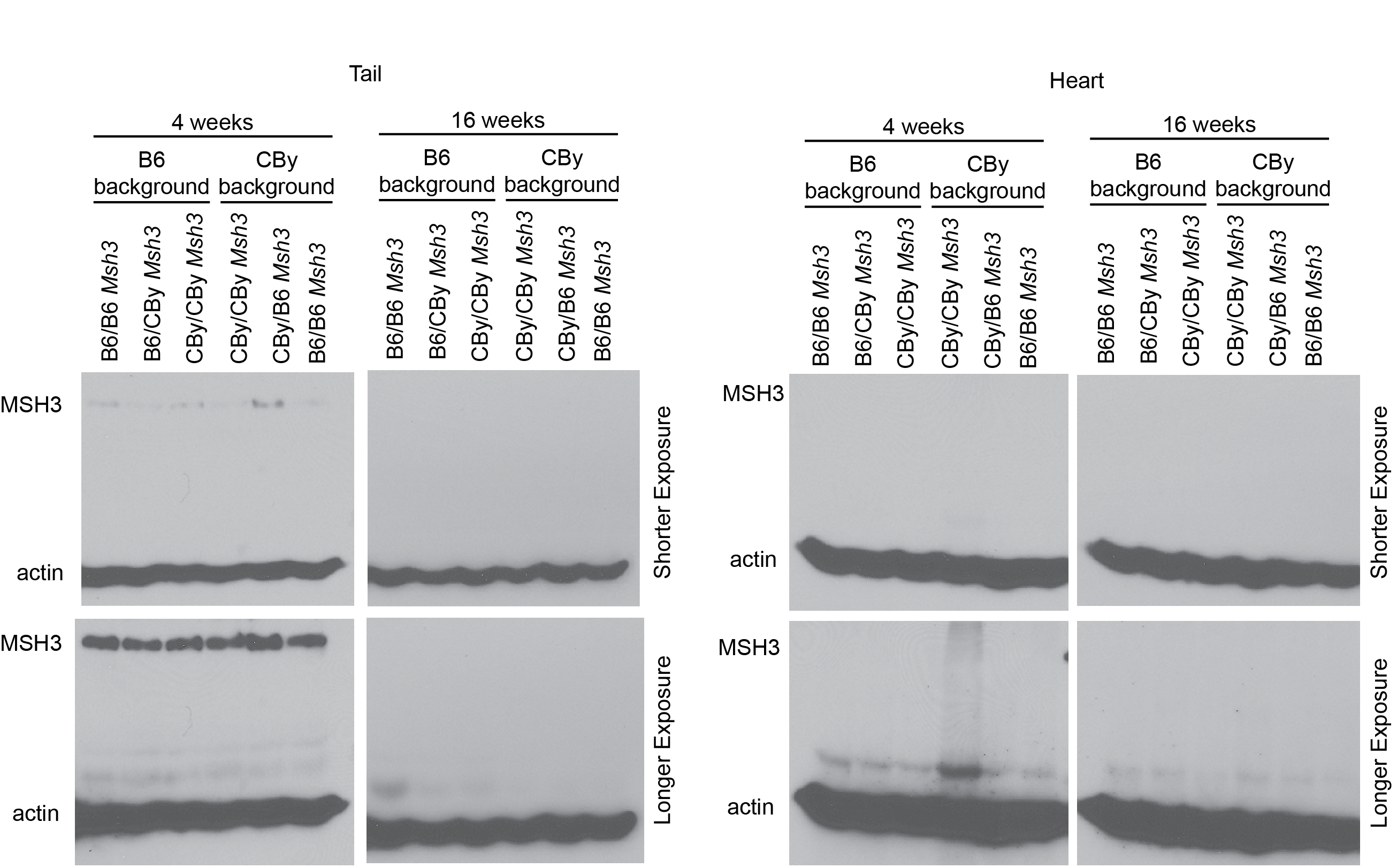

Supplement: Figure S5 — Western blot analysis of MSH3 protein level in heart and tail. MSH3 expression in tail and heart from 4 and 16 week-old mice. Western blot using only anti-MSH3 (Ab = 2F11) and actin antibodies in tail (left panel) and heart (right panel) from 4 and 16 week-old mice. Short exposure (top panel) and long exposures (bottom panel) are shown. MSH3 expression detected at low levels in tail of 4 week-old mice but not in 16 week-old mice. Undetectable levels of MSH3 in 4 and 16 week-old mice from heart tissue. MSH2 and MSH6 not detected in heart tissue of 4 and 16 week-old mice and low level detection of MSH2 in tail of 4 week-old (data not shown). (TIF) [file pgen.1003280.s005.tif]

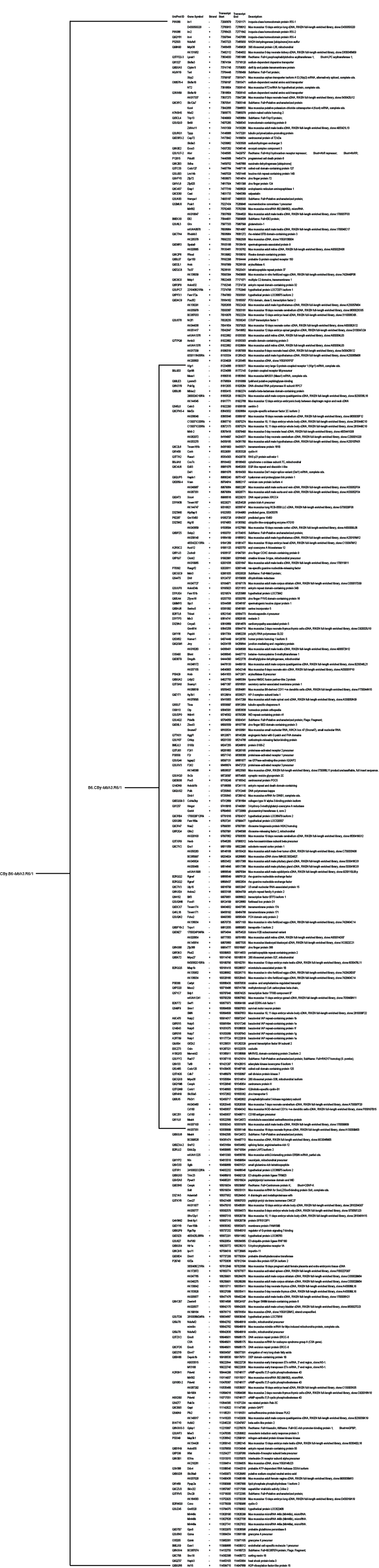

Supplement: Figure S7 — Contaminating genes flanking the Msh3 gene in the reciprocal congenics. The contaminating regions linked to the Msh3 gene in the reciprocal congenics. List representing Msh3-linked loci in the CBy.B6-Msh3 R6/1 and B6.CBy-Msh3 R6/1 reciprocal congenic mice. The contaminating regions linked to the Msh3 gene in the reciprocal congenics contain a limited number of genes, none of which have an obvious or documented role in CAG repeat instability. The regions linked to the Msh3 gene in the CBy.B6-Msh3 R6/1 reciprocal congenic mice span 43 Mbp and include 314 genes, of which 233 are protein-coding. In the B6.CBy-Msh3 R6/1 strain, the linked genes cover a region of approximately 22 Mbp, which lies within the 43 Mbp region of the CBy.B6-Msh3 R6/1 strain. A total of 151 genes are found within this region with 104-protein coding transcripts. (TIF) [file pgen.1003280.s007.tif]

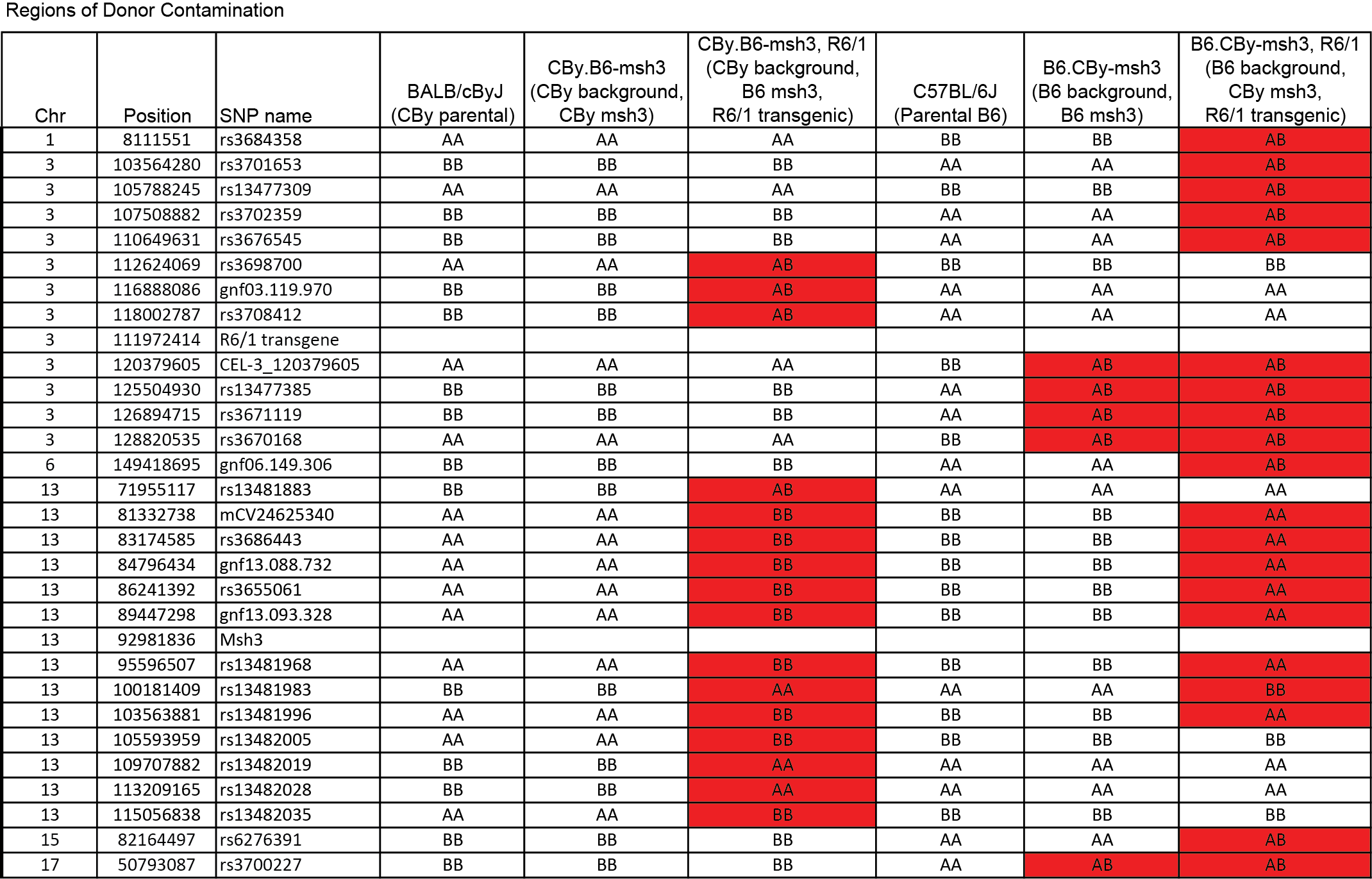

Supplement: Table S1 — SNP markers identified in the contaminating regions of both the congenic and reciprocal congenic mice listed with SNP marker name; chromosome number and position; R6/1 transgene and Msh3 gene integration and allelic representation of donor strain. Contaminating SNPs are highlighted in red. All B6 alleles are indicated with a B and all CBy alleles indicated with an A. (TIF) [file pgen.1003280.s008.tif]

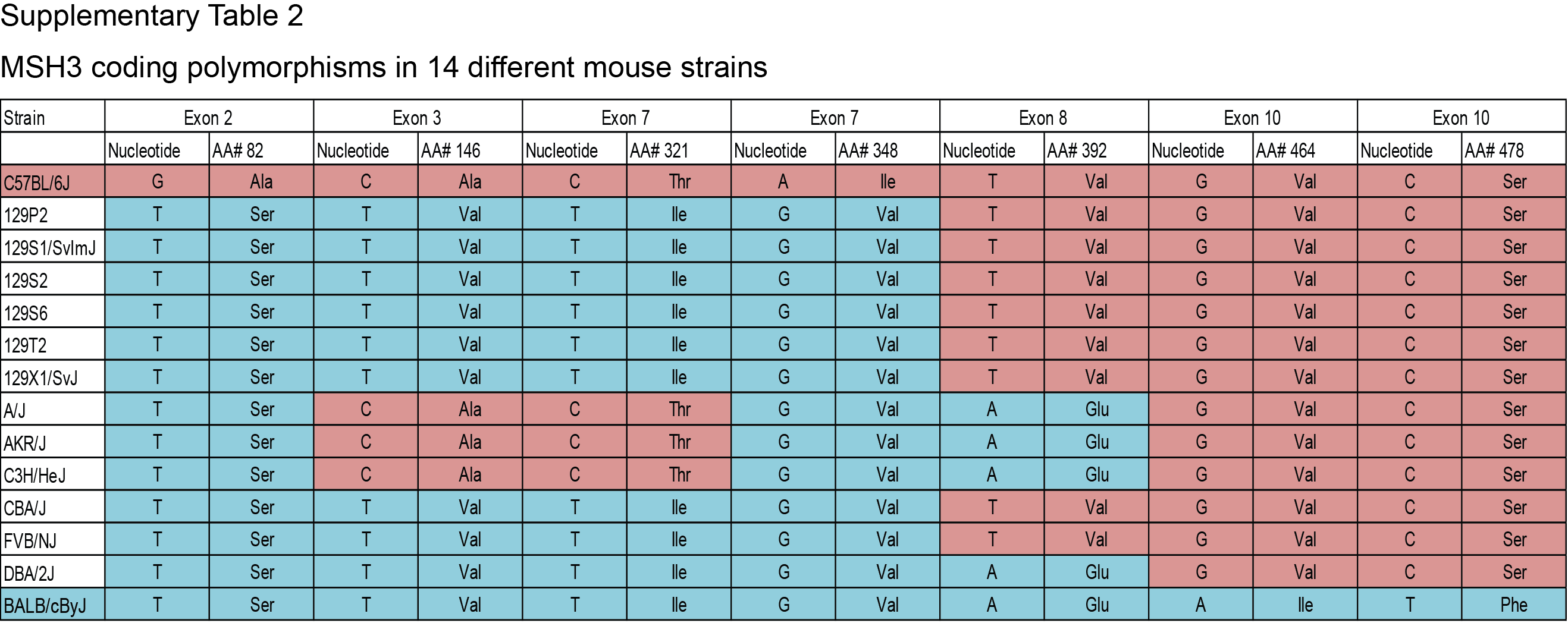

Supplement: Table S2 — MSH3 coding polymorphisms in 14 different mouse strains. MSH3 protein polymorphisms from C57BL/6 (B6) and BALB/CBy (CBy) mice. SNPs were identified or confirmed to those in dbSNP by sequencing the Msh3 gene, where similar amino acids were due to similar codons. In DBA/2J, exon 8, AA#392 was correctly identified to be T/Valine. For a given amino acid the same codon was used for the variants. (TIF) [file pgen.1003280.s009.tif]
